# Surface-Engineered 3D-Printed Polymeric Flat-Panel Photoreactors with P25 TiO_2_ Nanoparticles for Photocatalytic Hydrogen Evolution

**DOI:** 10.3390/nano16201283

**Published:** 2026-10-10

**Authors:** María José Torres, Jesús Román, María del Prado Garrido, Mireya Carvela

**Affiliations:** Hydrogen Technologies and Energy Transition Department, Association for Research and Technological Development of Industry in Castilla-La Mancha (ITECAM), Ctra. Pedro Muñoz, Km. 1, 13700 Tomelloso, Ciudad Real, Spain; mariajose.torres@itecam.com (M.J.T.); jesus.roman@itecam.com (J.R.); prado.garrido@itecam.com (M.d.P.G.)

**Keywords:** polymer nanocomposites, additive manufacturing, flat-panel flow photoreactor, photocatalytic hydrogen evolution, catalyst immobilization, surface engineering, computational fluid dynamics

## Abstract

In this work, a flat-panel photoreactor containing a serpentine channel was fabricated by LCD-based masked stereolithography and functionalized with commercial P25 TiO_2_. Computational fluid dynamics (CFD) confirmed laminar flow over 2–10 mL min^−1^ and described the velocity and wall-shear-stress distributions within the channel. Operating conditions were first established using suspended P25. Immobilization was then studied by varying binder type and content, P25 concentration and deposition method, together with pre-cure integration and direct printing of P25-loaded resin. The airbrushed P25/PARALOID™ B-72 coating provided the most favorable balance between reactor-level productivity and sustained response, with a post-maximum productivity retention of 47.8%. Direct printing reached the highest maximum apparent volumetric productivity (821.5 µmol H_2_,eq L_reactor_^−1^ h^−1^), corresponding to 1.7 and 2.1 times the values obtained with suspended and airbrushed configurations, respectively, but showed a much lower productivity retention of 12.9%. Post-mortem X-ray diffraction (XRD) and scanning electron microscopy–energy-dispersive X-ray spectroscopy (SEM–EDS) showed differences in P25 distribution and surface exposure among the tested configurations. These results show that the P25 incorporation strategy strongly affects both reactor-level productivity and operational stability.

## 1. Introduction

Fossil fuels have traditionally met most global energy demand, but their intensive use is associated with greenhouse-gas emissions and climate change. Global energy-related CO_2_ emissions increased by approximately 0.4% in 2025, reaching a new record of nearly 38.4 Gt CO_2_ and reinforcing the need for a rapid expansion of low-carbon energy systems [1]. Wind, solar and hydropower are already central to this transition [2]; however, the increasing contribution of variable renewable sources, particularly wind and solar, creates temporal mismatches between electricity generation and demand. Hydrogen (H_2_) can help address this limitation because it can be produced from renewable resources, stored, transported and subsequently converted into energy or used as a chemical feedstock. Its high gravimetric energy content (approximately 120 MJ kg^−1^ on a lower-heating-value basis and 142 MJ kg^−1^ on a higher-heating-value basis) further supports its use in applications for which batteries, grid connection or direct electrification are impractical [3].

Global hydrogen production is still dominated by fossil-fuel-based routes, particularly natural-gas reforming and coal gasification, which are associated with substantial CO_2_ emissions in the absence of carbon capture [4]. Water electrolysis provides a low-carbon alternative when powered by renewable electricity [5,6], and alkaline, proton-exchange-membranes and solid-oxide electrolyzers offer different combinations of cost, dynamic response, product purity and operating temperature [7,8,9]. Nevertheless, electrolysis requires an external electricity supply, power-conditioning equipment and water of a quality compatible with the technology [10]. These requirements motivate complementary solar–fuel routes that couple photon absorption and chemical conversion within the same process.

Photocatalytic H_2_ evolution is one such route. When a semiconductor absorbs photons with sufficient energy, electrons are promoted from the valence band to the conduction band, generating electron–hole pairs. If the photogenerated charge carriers reach the semiconductor surface before recombination, conduction-band electrons can participate in proton reduction, while valence-band holes drive oxidation reactions [11,12,13]. In the ideal overall water-splitting process, the half-reactions can be represented as follows:

Oxidation: H_2_O + 2H^+^ → 2H^+^ + ½O_2_ (1)

Reduction: 2H^+^ + 2e^−^ → H_2_ (2)

Overall reaction: H_2_O → H_2_ + ½O_2_ (3)

The overall reaction requires a minimum Gibbs free-energy change of 237 kJ mol^−1^, corresponding to a thermodynamic potential of 1.23 V under standard conditions. TiO_2_ is one of the most widely investigated photocatalysts because of its chemical stability, availability, comparatively low toxicity and suitable electronic structure [12,13,14]. Commercial P25 TiO_2_, which contains predominantly anatase together with a smaller rutile contribution, is frequently used as a benchmark material [15]. Its wide band gap restricts absorption predominantly to the UV region, while charge-carrier recombination further limits photocatalytic conversion efficiency.

In contrast to conventional water electrolysis, some photocatalytic H_2_-evolution schemes can intentionally exploit organic compounds present in aqueous streams as sacrificial electron donors or hole scavengers [16,17,18,19,20]. Suitable organic species can consume photogenerated holes and reduce charge recombination; however, water composition remains important because dissolved species may attenuate radiation, compete for active sites, generate undesired products or deactivate the photocatalyst [17,19]. Previous studies using commercial P25 as a benchmark photocatalyst have shown that the identity and chemical nature of the sacrificial electron donor can markedly influence the resulting H_2_ evolution, with alcohols, polyols and other organic substrates producing different photocatalytic responses under comparable irradiation conditions [21]. Photocatalytic H_2_ generation from organic substrates such as methanol has also been demonstrated with other oxide-based photocatalysts [22]. Formic acid is a useful model donor because both adsorbed formic acid and formate can undergo photooxidation on TiO_2_ [23]. In the present study, it was selected as a controlled sacrificial electron donor to evaluate how reactor operation and P25 deployment influence formic-acid-assisted gas evolution.

The use of formic acid also requires a clear distinction between photocatalytic reforming and overall water splitting. Formic acid can undergo dehydrogenation and dehydration through two limiting pathways [14,24,25]:

HCOOH → H_2_ + CO_2_ (4)

HCOOH → H_2_O + CO (5)

Dehydrogenation produces equimolar H_2_ and CO_2_, whereas dehydration yields CO and water. Previous studies on the photocatalytic conversion of formic acid over TiO_2_-based materials have experimentally identified H_2_, CO_2_ and CO among the gaseous products, with their relative contributions depending on the catalyst and reaction conditions [26,27]. Parallel reactions involving adsorbed formate, water, oxygen and reactive oxygen species may also contribute to the observed gas evolution [12,14,24]. Because gas composition was not determined, the measured gas volume was converted into a stoichiometric hydrogen equivalent (H_2_,eq) using the formic-acid dehydrogenation stoichiometry as a common reference for comparison among the different reactor configurations. H_2_,eq is therefore a comparative stoichiometric descriptor and does not represent directly measured H_2_ production or gas selectivity. The distinction is maintained throughout the manuscript, and the investigated process is not described as overall photocatalytic water splitting.

In addition to photocatalyst composition and reaction-medium chemistry, photoreactor engineering strongly affects the observed response. TiO_2_ is commonly employed as a slurry in laboratory photocatalytic reactors because dispersed nanoparticles provide high nominal surface area and short diffusion distances. Photocatalyst concentration often exhibits an optimum: excessive loading promotes aggregation, photon scattering and optical shielding [28,29,30]. Flow rate is similarly coupled to performance. Increased circulation can improve particle dispersion and external mass transfer but also shortens continuous flow residence time and modifies irradiation history. Recirculating-batch operation repeatedly exposes the reaction medium to the irradiated reactor, whereas continuous flow continuously introduces fresh solution; comparisons between the two modes should therefore account for residence time and cumulative liquid throughput rather than relying solely on operating time [28,30,31].

Although slurry operation establishes the attainable response of P25, catalyst recovery and control of nanoparticle release complicate repeated or continuous use. Immobilization avoids powder recovery and facilitates reactor reuse but can reduce the catalyst surface accessible to the liquid and incident radiation. The measured activity therefore reflects a balance among TiO_2_ loading, coating thickness, adhesion, porosity, wettability, irradiation and liquid–solid mass transfer [29,30,31]. Recent studies with immobilized P25 on porous supports further show that pore architecture and nanoparticle distribution can influence catalyst accessibility and the observed photocatalytic response, including under flow conditions [32,33]. Thick or binder-rich layers may increase optical attenuation and nanoparticle encapsulation, whereas insufficient binder can produce a highly exposed but mechanically weak coating. Photocatalytic hydrogen evolution has been demonstrated using polymer-supported Pt–TiO_2_ films, illustrating the feasibility of immobilization in a polymeric matrix [34]. However, retained TiO_2_ mass alone does not quantify the fraction that is both irradiated and accessible to reactants. Coating formulation and deposition methods must therefore be considered together. This trade-off is especially important for polymeric reactors, which cannot generally withstand the high-temperature annealing used for glass or metal substrates. Low-temperature, polymer-compatible formulations must consequently be evaluated in terms of initial activity, retention, reuse and post-mortem morphology. Acrylic matrices such as PARALOID™ B-72 have previously been used to immobilize TiO_2_ nanoparticles, including P25, in polymeric coatings [35,36]. Photocurable resins have likewise been employed as matrices for TiO_2_ immobilization and for the fabrication of photocatalytically active TiO_2_/polymer composites by vat photopolymerization [37,38,39].

Light delivery and reactor material impose additional constraints. Internal lamps can provide direct irradiation within the reaction volume, but suspended particles may cause strong light attenuation. External ultraviolet A (UVA) or visible-light sources simplify the optical arrangement and can reduce local heating, although the reactor window and catalyst layer then determine how much radiation reaches the active interface [40,41]. Quartz is commonly selected because of its broad optical transmission, thermal stability and chemical resistance [42,43,44]. Its cost, fragility, weight and limited manufacturability, however, hinder the rapid production of complex or customized flow architectures.

Flat-panel reactors offer alternative geometry with a high illuminated-area-to-volume ratio and a short optical path. Their planar arrangement can reduce dark regions and can be modularized for larger irradiated areas. Large-area particulate photocatalyst panels have demonstrated the potential of this concept for solar H_2_ production, while also showing that catalyst retention, optical utilization and durability must be addressed simultaneously [45,46]. These demonstrations concern overall water splitting with different photocatalysts and therefore support the planar reactor concept rather than providing direct performance benchmarks for the present formic-acid-assisted system. Replacing quartz with transparent polymeric components could reduce manufacturing complexity and enable integrated channels, provided that optical performance, chemical compatibility and stability under UVA irradiation are preserved.

Additive manufacturing expands this design space by producing tailored flow paths without conventional tooling. Fused deposition modelling is inexpensive and rapid, but interlayer voids, rough surfaces and limited optical quality may be problematic in photoreactors. Vat photopolymerization technologies, including stereolithography, digital light processing and liquid crystal display (LCD)-based masked stereolithography, generally provide high dimensional resolution and smooth surfaces and, when suitable transparent resins are used, can produce optically transparent components [39,47]. Photocatalytic functionality can be introduced after printing by depositing TiO_2_ onto the channel walls or during fabrication by dispersing it in the photocurable resin. Post-print coating favors catalyst exposure but requires adhesion under recirculation, whereas direct printing reduces assembly steps but may alter optical curing and encapsulate part of the active phase.

The method used to incorporate P25 determines the resulting polymer–TiO_2_ interface and, consequently, the fraction of catalyst exposed to the liquid and irradiation. Polymer composition and curing state, together with nanoparticle dispersion and localization, can affect catalyst attachment and stability during repeated use. In binder-assisted coatings, binder type, content and layer continuity also influence adhesion and optical attenuation. Depositions onto an incompletely cured surface may improve particle anchoring, whereas direct printing distributes P25 throughout the polymer matrix but may leave only part of the catalyst accessible at the surface [39,47]. These differences make the incorporation method an important factor in both photocatalytic performance and reactor stability.

Previous studies have demonstrated plastic flow reactors lined with P25-containing coatings [48] and stereolithography-printed TiO_2_/resin architectures [39] for pollutant degradation. These studies establish the feasibility of combining polymeric structures with photocatalytic functionality. A complementary question is how suspended P25 TiO_2_, binder-assisted coatings, deposition onto an incompletely cured polymer surface and direct printing compare within a common hydrodynamically characterized platform for formic-acid-assisted gas evolution. Addressing this question requires evaluation of catalyst exposure, retention and reactor-level productivity under clearly defined operating conditions.

In this work, a transparent polymeric flat-panel photoreactor containing a serpentine channel was manufactured by LCD-based masked stereolithography and functionalized with commercial P25 TiO_2_. Computational fluid dynamics first resolved the velocity and wall-shear-stress distributions over the investigated flow-rate range. Suspended-catalyst experiments then established the operating conditions and compared recirculating-batch and continuous flow operation, liquid volume and reactor reuse. TiO_2_ immobilization was subsequently evaluated by varying binder identity and content, photocatalyst loading and deposition method. Airbrushing and brushing were compared with two fabrication-integrated routes: deposition onto the incompletely cured polymer surface before final curing and direct printing of a TiO_2_-loaded resin. The selected configurations were assessed using H_2_,eq production and productivity-retention metrics, with apparent volumetric productivity calculated from the effective liquid volume of each configuration. Post-mortem X-ray diffraction and SEM–EDS provided structural and morphological evidence to support interpretation of TiO_2_ distribution and possible limitations in surface exposure. Unlike previous studies focused on individual fabrication or immobilization routes, this work compares suspended P25, binder-assisted coatings, pre-cure integration and direct printing within the same hydrodynamically characterized reactor platform. This approach enables comparison between surface-immobilized TiO_2_ coatings and a TiO_2_-containing polymer composite reactor, while examining the trade-offs among catalyst exposure, catalyst retention, productivity and reuse. The aim of this study was not to establish the intrinsic hydrogen-production performance of P25, but to assess how reactor fabrication and catalyst-integration strategies affect the performance of a polymeric photoreactor fabricated by LCD-based masked stereolithography, a common set of operating conditions.

## 2. Materials and Methods

### 2.1. Materials and Reagents

Commercial P25 TiO_2_ nanopowder (Evonik, Essen, Germany) was used as the photocatalyst without further treatment. Formic acid (98%, Labbox Labware S.L., Barcelona, Spain) was employed as the sacrificial electron donor and diluted with deionized water (conductivity: 2 µS cm^−1^) to prepare the 0.1 M aqueous reaction medium used throughout the study. A transparent UV-curable photopolymer resin (High Clear Resin, Anycubic, Shenzhen, China) was used for stereolithographic fabrication of the photoreactors and, where indicated, as the polymeric binder or matrix for TiO_2_ incorporation. PARALOID™ B-72 was selected as an alternative acrylic binder because of its well-established film-forming ability, adhesion, transparency and stability, and because TiO_2_-containing PARALOID B-72 composite coatings, including formulations with P25, have previously been reported [35,36]. Acetone (99.5%, Labbox Labware S.L., Barcelona, Spain) was used as the solvent in the coating formulations, whereas 2-propanol (99.9%, Labbox Labware S.L., Barcelona, Spain) was used for washing the printed components before post-curing.

### 2.2. Photoreactor Design and Additive Manufacturing

The photoreactor was designed using computer-aided design (CAD) software (SOLIDWORKS 2022 SP5.0) as a compact flat-panel flow cell containing a serpentine channel with seven straight sections connected by six 180° bends (Figure 1). The channel had a nominal width ($w_{c}$) of 6 mm, a height ($h_{c}$) of 3 mm and a center-to-center pitch (*p*) of 14 mm. Each straight section was 43 mm long, while the inlet and outlet ports had an internal diameter of 2.5 mm. The hydraulic path length, measured along the central channel in the CAD model, was 388.9 mm, and the nominal internal volume was 6.14 mL.

The reactor body and 2-mm-thick transparent window were manufactured separately by LCD-based masked stereolithography using an Anycubic Photon M3 Max printer (Anycubic, Shenzhen, China) and High Clear resin. The open-channel configuration facilitated removal of residual resin and provided access to the internal surfaces for TiO_2_ incorporation. The principal printing parameters are provided in Table S1.

After printing, the components were washed with 2-propanol and dried at room temperature. Except for the pre-cure configuration described in Section 2.3, the reactor components were post-cured under UVA irradiation at 60 °C for 15 min using a Form Cure V1 unit (Formlabs, Somerville, MA, USA). After the corresponding TiO_2_ incorporation procedure, the window was bonded to the reactor body using a thin layer of uncured High Clear resin and sealed by UVA irradiation for 15–30 min. The assembled reactor was subsequently connected to the recirculating experimental system through the inlet and outlet ports.

### 2.3. TiO_2_ Immobilization and Integration Strategies

Three approaches were investigated to incorporate P25 TiO_2_ into the polymeric photoreactor: binder-assisted post-print coating, immobilization before final curing and direct printing of a TiO_2_-loaded resin (Figure 2).

#### 2.3.1. Binder-Assisted Post-Print Coatings

For the binder-assisted coatings, P25 formulations were prepared using either PARALOID™ B-72 or High Clear resin as the binder and acetone as the liquid medium. These two polymeric matrices were selected to compare an acrylic binder previously employed in TiO_2_-containing coatings [35,36] with the photocurable resin used for reactor fabrication, thereby maintaining material compatibility with the printed substrate and enabling comparison with previously reported TiO_2_/photopolymer systems [38,39]. PARALOID™ B-72 was first dissolved in acetone under stirring at 35 °C for 1 h to accelerate dissolution and obtain a homogeneous binder solution. The High Clear resin formulation was prepared at room temperature without external heating. P25 TiO_2_ was subsequently added gradually to the corresponding binder solution and stirred until a homogeneous coating formulation was obtained. Binder type, binder loading (7.5, 15 and 30 wt.% on a TiO_2_-mass basis), TiO_2_ concentration (600, 1200 and 2400 mg L^−1^ of coating formulation), and deposition method were evaluated sequentially.

Approximately 15 mL of formulation was prepared for each reactor and deposited onto the internal channel surfaces by airbrush spraying or manual brush coating. For airbrush deposition, the formulation was transferred in successive portions to the airbrush reservoir using a Pasteur pipette and sprayed onto the internal channel surface using a 0.5 mm nozzle at a working pressure of approximately 3–4 bar. The remaining formulation was maintained under stirring and heating between successive spraying steps. Successive layers were applied until the channels were completely covered. Between successive applications, the coated reactor was exposed to an infrared (IR) lamp positioned approximately 50 cm from the reactor for 1 min, followed by approximately 5 min of drying without IR irradiation before application of the subsequent layer. Approximately five layers were applied by airbrushing and three by manual brushing. The nozzle-to-surface spraying distance and deposition rate were not quantitatively recorded during the manually operated airbrush procedure. The formulation containing 15 wt.% PARALOID™ B-72 and 1200 mg TiO_2_ L^−1^ was used for the subsequent comparison of deposition methods and reactor reuse.

The stability of the P25/PARALOID™ B-72 and P25/High Clear resin coatings was additionally assessed under separate chemical and irradiation conditions. Coated reactors were subjected to 24 h of recirculation with 0.1 M formic acid in the absence of irradiation and, separately, to deionized water under UVA irradiation. Reactor mass was recorded before and after exposure, and macroscopic changes in the coatings and polymeric reactor body were assessed.

#### 2.3.2. Pre-Cure P25/Resin Integration

For pre-cure integration, the printed reactor was washed and dried, but the conventional post-curing step was omitted. A TiO_2_-containing resin formulation (15 mL of a TiO_2_/resin formulation containing 1200 mg L^−1^ TiO_2_) was applied by brush directly onto the incompletely cured channel surface. The reactor was subsequently irradiated with UVA light for 15 min to complete polymerization and fix the catalytic layer. Thus, unlike conventional post-print brush coating, the residual uncured resin at the reactor surface acted as the binding phase.

#### 2.3.3. Direct Printing of TiO_2_-Loaded Resin

For direct printing, P25 TiO_2_ was dispersed at 1200 mg L^−1^ in the High Clear resin under mechanical stirring for 1 h before transfer to the printer vat. The spatial dispersion of P25 within the resin and possible sedimentation during the printing period were not quantitatively characterized. The direct incorporation of TiO_2_ into photocurable resins has previously been demonstrated for the fabrication of photocatalytically active polymer–TiO_2_ composites by vat photopolymerization, including systems based on commercial P25 [38,39]. The formulation was transferred to the printer vat and used to fabricate the complete reactor. After printing, the component was washed with 2-propanol, dried and post-cured under UVA irradiation at 60 °C for 15 min.

Following TiO_2_ incorporation, the residual channel height ($h_{c,eff}$) was measured at three different points using a digital caliper, and the mean value was used as a geometrical descriptor of the available channel height after catalyst incorporation. The equivalent layer thickness ($\delta_{eq})$ was calculated as the difference between the nominal channel height of 3.00 mm and $h_{c,eff}$. The configuration-specific effective liquid volume ($V_{R,eff}$) used for reactor-performance normalization was experimentally determined for each assembled reactor configuration, as described in Supplementary Note S1.2, and was treated independently from $h_{eff}$ and the nominal CAD geometry used in the CFD analysis. Because catalyst incorporation reduced the available channel height, the different configurations contained different liquid volumes within the irradiated reactor region. The configuration-specific effective reactor volume was therefore used to normalize reactor performance on a common volumetric basis. This normalization accounts for the geometrical changes introduced by each catalyst-deployment strategy and does not represent the intrinsic photocatalytic activity of P25. The experimentally determined effective liquid volumes were not used to define the CFD domains. The preliminary CFD analysis retained the nominal 6 mm × 3 mm CAD geometry, whereas the additional configuration-specific simulations used the representative residual channel heights to assess the hydrodynamic effect of channel constriction. The calculation of the effective reactor volume and apparent volumetric productivity is described in Supplementary Note S1.2, and the resulting reactor-level performance metrics are reported in Table 1. Applied, retained and nominally incorporated TiO_2_ masses, together with the mass basis used for normalization, were determined as described in Supplementary Note S1.2 and are summarized in Table S8.

### 2.4. Computational Fluid Dynamics (CFD)

The hydrodynamic behavior of the serpentine photoreactor was investigated using SOLIDWORKS Flow Simulation 2022 SP5.0. A three-dimensional, steady-state internal-flow model was generated from the CAD geometry described in Section 2.2. Water at 293.2 K was used as a hydrodynamic approximation of the dilute 0.1 M aqueous formic-acid solution. The liquid was treated as incompressible and Newtonian, with no-slip, hydraulically smooth channel walls. Heat transfer was not included in the CFD model. However, the circulating-liquid temperature, monitored experimentally as described in Section 2.5, remained below 30 °C under the irradiation conditions investigated. The model was therefore retained as an isothermal hydrodynamic description. Gravitational effects and free-surface phenomena were also neglected.

Volumetric flow rates of 2, 4, 6, 8 and 10 mL min^−1^ were imposed normal to the inlet, and atmospheric static pressure (101,325 Pa) was specified at the outlet. The rectangular channel had a hydraulic diameter of 4.0 mm. The corresponding Reynolds numbers ranged from approximately 7.4 to 36.9, confirming laminar flow throughout the interval investigated. The theoretical hydrodynamic parameters and their calculation are provided in Supplementary Note S1 and Table S5.

The computational domain was discretized using the Cartesian mesh implemented in SOLIDWORKS Flow Simulation. Mesh sensitivity was assessed at 4 mL min^−1^ using resolution levels 5, 6 and 7, corresponding to 112,713; 311,013 and 855,852 fluid cells, respectively. Because the global velocity and surface-averaged wall-shear-stress results remained effectively unchanged between successive refinements, resolution level 6 was selected for the final simulations. The complete mesh-independence assessment is reported in Table S2.

Convergence was monitored using the inlet and outlet volumetric flow rates, average and maximum velocities, static pressures and average wall shear stress. All final simulations met the solver convergence criteria, with an inlet–outlet flow imbalance below 0.002%. Velocity fields, flow trajectories, pressure and wall-shear-stress distributions were evaluated throughout the reactor. Transverse axial-velocity profiles were extracted from a common straight-channel section immediately upstream of a bend. Nominal CFD residence time $\left(\tau_{CFD}\right)$ and pressure drop were calculated from the nominal internal reactor volume, flow rate and area-averaged inlet and outlet pressures, as described in Supplementary Note S1. Additional steady-state CFD simulations were performed at 4 mL min^−1^ using simplified configuration-specific geometries with channel heights of 3.00, 1.68, 1.80 and 1.23 mm for the suspended-P25, airbrushed P25/PARALOID™ B-72, pre-cure P25/resin and directly 3D-printed TiO_2_/resin configurations, respectively. All other boundary conditions were maintained, assuming smooth walls and uniform channel heights.

### 2.5. Experimental Setup and Operating Procedure

Photocatalytic experiments were conducted in a closed, ventilated irradiation chamber to maintain a reproducible lamp-to-reactor geometry and limit stray UVA radiation. The recirculating system comprised a feed reservoir, a peristaltic pump (Percom N-M II, JP Selecta, Barcelona, Spain), the flat-panel photoreactor, a gas–liquid separator and a 25 mL water-displacement gas meter (Figure 3). The reactor was positioned horizontally on an adjustable platform. The feed reservoir was positioned away from the UVA source and remained covered throughout the experiments to prevent direct irradiation of the reaction medium contained in the reservoir. Photographs of the complete experimental installation and the assembled photoreactor are provided in Figure S1.

Before irradiation, the initial gas volume present in the experimental system was recorded and subsequently accounted for in the calculations. The gas–liquid separation system included a dedicated purge line equipped with a valve. During photocatalytic operation, the purge valve was opened briefly (approximately 1–2 s) between measurement intervals and was then closed again; no external purge gas was introduced. Gas-production rates were determined from discrete measurement intervals initiated after each purge and were therefore not calculated from a continuously accumulated gas volume. Dissolved-oxygen concentration in the liquid phase was not quantitatively measured, and complete removal of dissolved O_2_ cannot therefore be confirmed.

The photocatalytic system was operated in two configurations: recirculating-batch and single-pass continuous-flow modes. For recirculating-batch operation, the 0.1 M formic-acid solution was pumped from the reservoir through the photoreactor and subsequently directed to the gas–liquid separator. The separated liquid was continuously returned to the reservoir. For continuous-flow operation, freshly prepared 0.1 M formic-acid solution was continuously supplied from the feed reservoir and passed through the photoreactor only once. The liquid effluent leaving the gas–liquid separator was collected in a separate vessel and was not recirculated. These experiments were conducted for 3 h at each investigated flow rate, and the required feed volume was prepared according to the corresponding flow rate and operating time. In both operating modes, the gas separated from the liquid was transferred to two graduated columns hydraulically connected at their bases. Gas entering the measuring column displaced water towards the pressure-balancing column, and the displaced volume was recorded at predetermined sampling times. This system consisted of 25 mL graduated columns with a volumetric resolution of 1 mL, which were volumetrically verified before use. Before each photocatalytic experiment, the complete gas-measurement system was checked by introducing a known air flow, independently measured at the inlet, and comparing it with the corresponding displaced volume. Water was used exclusively as the displacement liquid and did not enter the photocatalytic circuit. The evolved gas may contain species with different aqueous solubilities; therefore, the composition of the gas generated in the reactor is not necessarily preserved quantitatively in the volume collected by water displacement. In particular, CO_2_ can partition into the aqueous phase [49]. No correction for dissolved CO_2_ was applied. Consequently, the displacement measurement was used as an operational measure of gas evolution rather than as a direct quantitative determination of individual gaseous species. The displaced gas volume constituted the primary experimental measurement. To compare the different operating conditions on a molar basis, the recorded cumulative volume was first converted into total evolved-gas moles using the ideal-gas equation. Because the gas composition was not determined chromatographically, the resulting amount was subsequently expressed as a stoichiometric hydrogen equivalent, $\eta_{H_{2,eq}}$, based on the formic-acid dehydrogenation pathway defined in Equation (4).

The cumulative amount of evolved gas ($\eta_{gas}$) was calculated using the ideal gas equation:

(6) $$\eta_{gas}(t)=\frac{P_{g}V_{g}(t)}{{RT}_{g}}$$

where *P*_g_ is the local atmospheric pressure, *V*_g_ is the cumulative displaced volume, R is the ideal-gas constant and *T*_g_ is the recorded gas temperature. Water-vapor pressure was not corrected for in the calculation. Based on the dehydrogenation stoichiometry of formic acid shown in Equation (4), the stoichiometric hydrogen-equivalent amount was defined as:

(7) $$\eta_{H_{2,eq}}\left(t\right)=0.5\eta_{gas}\left(t\right)$$

Accordingly, $\eta_{H_{2,eq}}$ represents a stoichiometric equivalent derived from the total evolved gas and not directly quantified hydrogen. These definitions were applied consistently to all suspended and immobilized catalyst configurations. The additional calculations used to obtain interval production rates, mass-specific rates, apparent volumetric productivities and productivity-retention metrics are provided in Supplementary Note S1.2.

UVA irradiation was supplied by lamps with nominal electrical powers of 1, 9 and 15 W and emission wavelengths of 385–395 nm. Unless otherwise stated, the experiments were performed using the 9 W source positioned 5 cm above the reactor window. An additional lamp-to-reactor distance of 21 cm was evaluated using the 9 W source to assess the effect of irradiation geometry at a lower incident irradiance. Temperature was monitored at 5 min intervals using a thermometer, both at the external surface of the photoreactor, to assess the thermal exposure of the polymeric material, and in the circulating reaction liquid, to evaluate possible temperature-induced changes in the fluid properties. Both temperatures remained below 30 °C throughout the photocatalytic experiments. Additional monitoring experiments were performed under representative photocatalytic operating conditions to assess the evolution of pH during irradiation. The pH of the circulating reaction medium was measured at 15 min intervals using a benchtop pH meter (Hanna Instruments, Woonsocket, RI, USA). Incident and transmitted irradiances were measured using an SM206-SOLAR radiometer (Sanpometer, Shenzhen, China) at the reactor plane and after passage through the corresponding reactor configuration, respectively. These measurements were performed at discrete measurement positions and were not intended to provide a full spatial irradiance map of the reactor surface. The apparent irradiance transmission ratio (T_I_) was calculated as:

(8) $$T_{I}=\frac{q_{out}^{\prime \prime }}{q_{inc}^{\prime \prime }}\times 100$$

where $q_{inc}^{\prime \prime }$ and $q_{out}^{\prime \prime }$ are the irradiances measured before and after passage through the reactor assembly, respectively. This ratio was used only as an optical descriptor of the complete reactor assembly and was not interpreted as absorbed-photon efficiency, quantum yield or photocatalytic conversion efficiency. Although incident irradiance was measured and used to calculate the corresponding radiant-energy input, the incident photon flux was not directly determined from spectrally resolved measurements. Therefore, an apparent quantum yield was not calculated.

For suspended-catalyst experiments, the required amount of P25 was added to the 0.1 M formic-acid solution to obtain concentrations of 50, 100 or 200 mg L^−1^. The suspension was stirred for 30 min before irradiation to promote P25 dispersion and was maintained under continuous stirring in the reservoir throughout the experiment. Liquid flow rates of 2–10 mL min^−1^ and initial liquid volumes of 25, 50, 100 and 200 mL were evaluated using suspended P25 as part of the preliminary assessment of the operating conditions. For experiments with immobilized or fabrication-integrated TiO_2_, no suspended P25 was added to the reaction medium. Unless otherwise specified, these experiments were conducted at 4 mL min^−1^ using 25 mL of 0.1 M formic-acid solution. Given that the time required to circulate a liquid volume equivalent to the initial liquid inventory depends on both flow rate and initial volume, operating progress was expressed as the nominal number of recirculation cycles (N) rather than operating time alone. The number of recirculation cycles was calculated as:

(9) $$N=\frac{Q_{t}}{V_{0}}$$

where *Q* is the liquid flow rate, t is the operating time and *V*_0_ is the initial liquid volume. Initial screening experiments were carried out for 10 nominal recirculation cycles. After selection of the reference operating conditions, selected immobilized and fabrication-integrated configurations were operated for longer periods to evaluate the evolution of the photocatalytic response until an approximately stable rate regime was observed. To assess short-term repeatability, the experiments at 4, 8 and 10 mL min^−1^ were initially performed in duplicate using the same uncoated photoreactor under otherwise identical conditions. For the selected flow rate of 4 mL min^−1^, two additional replicate runs were performed under the same conditions using the same uncoated photoreactor and freshly prepared reaction solution, resulting in a total of four runs at this condition. The four individual profiles obtained at 4 mL min^−1^ are reported in Figure S7. Reactor-to-reactor reproducibility of the selected airbrushed P25/PARALOID™ B-72 configuration was assessed using two independently prepared coated reactors under identical operating conditions (Figure S8a). Sequential reuse was evaluated separately through three runs performed with the same airbrushed reactor after washing and replacement of the reaction solution (Figure S8b). Longer experiments used to assess sustained operation are identified separately in the corresponding figures. The complete experimental matrix is provided in Table S3.

### 2.6. Post-Mortem Characterization

After photocatalytic operation, representative sections were cut from the channel regions of four reactor configurations: directly printed P25/resin reactor, pre-cure P25/resin integration, airbrushed P25/PARALOID™ B-72 and an uncoated reactor previously operated with suspended P25.

X-ray diffraction (XRD) patterns were recorded using a Philips PW-1711 diffractometer (Philips Analytical, Almelo, The Netherlands) with Cu Kα radiation (λ = 1.5418 Å) at 40 kV and 40 mA. Data were collected over 20–60° 2θ using a step size of approximately 0.0204°. The patterns were background-corrected, independently normalized and vertically offset for comparison. Phase identification was performed qualitatively using reference diffraction data. Phase fractions, crystallinity and crystallite sizes were not calculated because the dominant polymeric contribution and the low TiO_2_ diffraction signal prevented reliable quantitative analysis.

Surface morphology and elemental distribution were examined by scanning electron microscopy (SEM) using a JEOL JSM-6490LV scanning electron microscope (JEOL Ltd., Akishima, Tokyo, Japan) equipped with secondary-electron, backscattered-electron and energy-dispersive X-ray spectroscopy (EDS) detectors (Oxford Instruments NanoAnalysis, High Wycombe, UK). Analyses were conducted at an accelerating voltage of 20 kV after applying a conductive Au/Pd coating. SEM was used to examine surface morphology, while EDS and Ti elemental mapping were used to evaluate local elemental composition and titanium distribution. Au and Pd signals originating from the conductive coating were excluded from the compositional discussion. SEM imaging and Ti Kα elemental mapping were examined in two independent regions of each reactor specimen. The additional mapped regions are provided in Figure S11.

## 3. Results and Discussion

### 3.1. Hydrodynamic Assessment of the Serpentine Photoreactor

The hydrodynamic behavior of the serpentine photoreactor was evaluated over the experimental flow-rate range of 2–10 mL min^−1^. Based on the nominal 6 × 3 mm channel cross-section and the properties of water at 293.2 K, the Reynolds number increased from 7.4 to 36.9, confirming laminar flow under all conditions investigated (Table S5). Over the same interval, the nominal CFD residence time decreased from approximately 184 s at 2 mL min^−1^ to 37 s at 10 mL min^−1^. Increasing the flow rate therefore enhanced liquid renewal while shortening the time spent within the irradiated channel during each pass. The hydrodynamic behavior of the serpentine photoreactor was evaluated over the experimental flow-rate range of 2–10 mL min^−1^ (Figure 4).

Figure 4a shows the velocity distribution and flow trajectories at 4 mL min^−1^, selected as a representative operating condition. The trajectories followed the complete serpentine path without extensive flow separation or large stagnant regions. A higher-velocity core developed away from the walls, while the velocity approached zero at the solid boundaries. The velocity distribution remained comparatively uniform along the straight sections and was locally redistributed at the 180° bends because of flow redirection and channel curvature, as commonly observed in rectangular curved channels [50]. At 4 mL min^−1^, the nominal bulk velocity calculated from the channel cross-sectional area was 3.70 × 10^−3^ m s^−1^. The wall-normal velocity profile extracted immediately upstream of a representative bend reached approximately 5.5 × 10^−3^ m s^−1^, whereas the maximum displayed over the complete domain was approximately 6 × 10^−3^ m s^−1^. This difference reflects the use of a single straight-channel section for profile extraction, while the domain maximum may occur at a bend or geometrical transition. Increasing the flow rate raised the velocity magnitude while preserving the overall spatial distribution (Figure S2).

The profiles in Figure 4c exhibited a broad central maximum and approached zero at both walls, consistent with laminar no-slip flow. The local maximum increased from approximately 2.5 × 10^−3^ m s^−1^ at 2 mL min^−1^ to 12.1 × 10^−3^ m s^−1^ at 10 mL min^−1^, while the profile shape remained approximately self-similar. Although the channel bends redistributed the velocity field, the low Reynolds numbers and the absence of pronounced secondary-flow structures in the present CFD results suggest that curvature-induced secondary motion remained limited under the evaluated conditions [50].

Wall shear stress was comparatively uniform along the straight sections but increased locally near the inner regions of the bends, where flow redirection increased the near-wall velocity gradient (Figure 4b). The supplementary distributions show that increasing the flow rate increased the wall shear stress without substantially changing the location of the higher-stress regions (Figure S3). Although the predicted wall shear stress remained low, CFD alone cannot establish coating stability, which also depends on binder adhesion, curing, layer thickness and repeated wetting. For example, Zhou et al. observed a clear dependence of photocatalytic performance on flow conditions in 3D-printed P25-coated reactors [48], while Li et al. related the operation of TiO_2_-coated optofluidic reactors directly to the imposed residence time [51]. CFD studies of immobilized serpentine photoreactors have likewise shown that flow-path design influences fluid distribution, residence time and contact with the catalytic surface [52]. Coating stability was therefore evaluated experimentally through reuse tests and post-mortem characterization. The hydrodynamic effect of catalyst-induced channel constriction was evaluated at 4 mL min^−1^ using configuration-specific simplified geometries (Figures S4 and S5). The maximum local velocity increased from 0.0067 m s^−1^ for suspended P25 to 0.0148, 0.0132 and 0.0212 m s^−1^ for the airbrushed, pre-cure and directly 3D-printed configurations, respectively. The corresponding maximum wall shear stress increased from 0.0106 Pa to 0.0422, 0.0361 and 0.0906 Pa, with the largest effect observed for the directly printed reactor, which had the smallest residual channel height. These simulations represent idealized smooth-wall conditions and do not account for local coating roughness or thickness heterogeneity.

Overall, the nominal-channel CFD confirmed laminar flow with low wall shear stress, while the configuration-specific simulations showed that channel constriction modifies local velocity and shear stress. Therefore, the nominal hydrodynamic results should not be directly extrapolated to the catalyst-containing configurations, and the operating flow rate was ultimately selected experimentally.

### 3.2. Evaluation of Operating Conditions and Reactor Configuration Using Suspended P25

Suspended P25 was first used to establish photocatalytic operating conditions and subsequently to evaluate the influence of reactor operating mode, initial liquid volume and cumulative use. As the gas composition was not determined, all quantitative comparisons are reported as stoichiometric hydrogen equivalents (H_2_,eq), as defined in Section 2.5. These values are used as comparative reactor-performance metrics and should not be interpreted as directly quantified H_2_ production or gas selectivity.

#### 3.2.1. Photocatalytic Operating-Condition Screening

The operating conditions were established sequentially using suspended P25 (Figure 5). Because gas composition was not analyzed, all results are reported as stoichiometric hydrogen equivalents (H_2_,eq) as defined in Section 2.5.

Prior to operating-condition screening, control experiments were performed to assess background gas evolution (Table S4). Only minor accumulated gas signals were observed for water + UVA (0.077 µmol), 0.1 M HCOOH + UVA (0.032 µmol), and P25 + 0.1 M HCOOH without UVA (0.026 µmol). As these controls represent different physicochemical conditions rather than a common instrumental blank, their values were not subtracted from the photocatalytic datasets.

Adding 0.1 M formic acid enhanced gas evolution over the complete flow-rate range (Figure 5a). At 4 mL min^−1^, the average total gas-evolution rate increased from approximately 1.2 to 4.6 µmol gas h^−1^, corresponding to a value approximately 3.8 times that obtained without formic acid. This response is consistent with formic acid acting as an electron donor and hole scavenger, thereby reducing charge-carrier recombination and favoring reduction reactions over TiO_2_ [26,53].

The liquid flow rate produced a non-monotonic response (Figure 5b). At 25 min, the highest specific rate was obtained at 4 mL min^−1^, reaching 1267 µmol H_2_,eq g^−1^ TiO_2_ h^−1^ and exceeding the remaining conditions by 36.6–138.6%. The same highest response among the tested flow rates was observed after 10 recirculation cycles, with a rate of 1877 µmol H_2_,eq g^−1^ TiO_2_ h^−1^. This result indicates a balance between liquid renewal, particle redistribution and irradiation time per pass. The complete time-resolved profiles are provided in Figure S6. Short-term repeatability was assessed at 4, 8 and 10 mL min^−1^. Duplicate experiments reproduced the overall flow-rate-dependent response, with 4 mL min^−1^ consistently providing the highest response among the conditions tested. Since 4 mL min^−1^ was subsequently selected as the reference operating condition, two additional replicate runs were performed under the same conditions and included in the repeatability assessment. The four individual profiles obtained at 4 mL min^−1^ are shown in Figure S7. The final H_2_,eq production rate at N = 10 for the four runs was 4.7 ± 1.3 µmol H_2_,eq h^−1^ (mean ± SD, *n* = 4), indicating appreciable run-to-run variability in the absolute production rate. Therefore, these results are interpreted as a short-term repeatability assessment rather than as a comprehensive statistical characterization of experimental uncertainty.

Among the tested catalyst loadings, 100 mg L^−1^ provided the highest response (Figure 5c). At 100 mg L^−1^ P25, the production rate increased to approximately 4.7 µmol H_2_,eq h^−1^, whereas final values of approximately 0.7 and 0.4 µmol H_2_,eq h^−1^ were obtained at 50 and 200 mg L^−1^, respectively. The lower response at 50 mg L^−1^ is consistent with the lower amount of photocatalyst available within the irradiated suspension, whereas the decrease at 200 mg L^−1^ is consistent with increased particle aggregation, light scattering and optical screening [54]. The acidic medium may also affect catalyst dispersion and interfacial adsorption. The theoretical pH of 0.1 M formic acid is approximately 2.4, below the reported point of zero charge of P25 TiO_2_, and the surface would therefore be expected to be predominantly protonated [55]. Additional pH measurements confirmed that the formic-acid-containing media remained acidic under the investigated photocatalytic and control conditions. In a representative P25/0.1 M formic-acid experiment under UVA irradiation, the pH changed only moderately from 2.30 to 2.64. Zeta potential was not measured during operation. Consequently, the contribution of surface charge cannot be demonstrated experimentally, and the reduced response at 200 mg L^−1^ is conservatively interpreted in terms of aggregation and radiative attenuation [23].

Nominal lamp power was not proportional to the energy-normalized response (Figure 5d). Because the incident photon flux was not directly determined from spectrally resolved measurements, the energy-normalized H_2_,eq yield should be interpreted as an operational reactor-performance metric rather than as an apparent quantum yield or intrinsic photochemical efficiency. The 1 and 9 W sources produced 28.8 and 29.3 µmol H_2_,eq kJ_inc^−1^, respectively, whereas the response decreased to 3.1 µmol H_2_,eq kJ_inc_^−1^ with the 15 W source. At a common cumulative incident energy input of approximately 0.3 kJ L^−1^, increasing the lamp-to-reactor distance from 5 to 21 cm increased cumulative production from 13.7 to 18.6 µmol H_2_,eq L^−1^. The reactor response therefore depended on irradiance distribution and exposure conditions rather than nominal electrical power alone [56]. Based on this screening, 0.1 M formic acid, 100 mg L^−1^ P25, 4 mL min^−1^ and 9 W UVA irradiation were selected for subsequent experiments.

#### 3.2.2. Operating Mode, Reaction Volume and Reactor Reuse

Under the selected photocatalytic conditions, recirculating-batch and continuous flow operation, initial liquid volume and cumulative reactor use were subsequently evaluated (Figure 6).

Recirculating-batch operation reached a maximum specific rate of 2008 µmol H_2_,eq g^−1^ TiO_2_ h^−1^, 53.9% higher than the 1305 µmol H_2_,eq g^−1^ TiO_2_ h^−1^ obtained in continuous flow operation (Figure 6a). The time-averaged specific rate was also approximately 3.4 times that obtained under continuous-flow operation. The higher response under recirculating-batch operation is consistent with repeated passage of the suspension through the irradiated channel, despite the short nominal CFD residence time per pass of approximately 1.53 min. Increasing the initial liquid volume reduced the reactor response (Figure 6b). The fraction of liquid contained within the illuminated reactor decreased from 24.6% at 25 mL to 3.1% at 200 mL, while the time required for one nominal recirculation cycle increased from 6.25 to 50 min. The higher response obtained with 25 mL is therefore consistent with more frequent processing of the total liquid inventory through the irradiated reactor volume. The uncoated reactor remained operational over three successive experiments and 270 min of cumulative use (Figure 6c). A quasi-steady rate of approximately 3.8 µmol H_2_,eq h^−1^ was observed between 90 and 150 min, followed by a decrease to 2.6 µmol H_2_,eq h^−1^. Because the suspension was renewed and its composition differed among experiments, this profile cannot be interpreted as an intrinsic deactivation curve. Nevertheless, it demonstrates continued operability of the printed reactor over 4.5 h of cumulative experimental use. Recirculating-batch operation with an initial volume of 25 mL was therefore selected for the subsequent immobilization experiments.

### 3.3. Evaluation and Operational Stability of Immobilized P25 Coatings

After establishing the reference conditions with suspended P25, the coating formulation was systematically evaluated to identify conditions providing a favorable balance between catalyst accessibility, retention and operational stability (Figure 7).

Binder selection strongly affected the photocatalytic response (Figure 7a). The PARALOID™ B-72 coating clearly outperformed the photocurable-resin coating, reaching 114.3 µmol H_2_,eq g^−1^ TiO_2_ after 120 min, compared with 36.1 µmol H_2_,eq g^−1^ TiO_2_ for the resin-based formulation. This higher response is consistent with greater P25 accessibility in the PARALOID™ B-72 coating, whereas the cured resin may have restricted catalyst exposure through partial encapsulation and associated optical or mass-transfer limitations [35,36]. The stability of both binder systems was additionally examined under separate 24 h chemical- and UVA-exposure conditions. Apparent whole-reactor mass variations ranged from −0.294% to +1.18%, depending on the coating and exposure condition. However, small residual liquid droplets remained on the internal channel surfaces after the drying procedure and could not be completely removed. Consequently, these whole-reactor mass variations were not interpreted quantitatively as polymer degradation, coating loss or catalyst release. Macroscopic inspection before and after the exposure tests did not reveal visible cracking, swelling or deformation of the High Clear resin reactor body. No clear additional discoloration of the polymeric reactor or evident large-scale coating delamination was observed. These observations indicate preservation of the macroscopic integrity of the reactor over the investigated exposure period; however, chemical or microstructural changes not detectable by visual inspection cannot be excluded. Nevertheless, prolonged irradiation of TiO_2_-containing acrylic coatings can promote photo-oxidative modification of the polymer matrix through photocatalytically generated reactive species [35,36,57]. Therefore, the present results should be considered a short-term stability assessment rather than evidence of long-term photochemical durability.

A non-monotonic dependence on both PARALOID™ B-72 content and P25 concentration was observed (Figure 7b). At 1200 mg L^−1^ P25, the maximum H_2_,eq production rate increased from 0.5 to 1.2 µmol H_2_,eq h^−1^ between 7.5 and 15 wt.% (on a TiO_2_-mass basis) binder, but decreased to 0.264 µmol H_2_,eq h^−1^ at 30 wt.%. This decrease may be associated with reduced catalyst accessibility at higher polymer content, although catalyst accessibility was not directly quantified. At the selected 15 wt.% PARALOID™ B-72 content, increasing P25 from 600 to 1200 mg L^−1^ enhanced the rate from 0.38 to 1.17 µmol H_2_,eq h^−1^, whereas 2400 mg L^−1^ reduced it to 0.09 µmol H_2_,eq h^−1^. Possible explanations include increased particle aggregation and light attenuation within the coating; however, these mechanisms were not directly characterized in the present study and should therefore be considered hypotheses. The narrow deposition-yield range (63.1–75.1%) shows that the observed performance differences cannot be explained solely by the retained TiO_2_ mass and may also reflect differences in catalyst accessibility and spatial distribution.

Deposition method further affected the photocatalytic response of the immobilized catalyst (Figure 7c). Airbrushing reached a maximum specific rate of 101.2 µmol H_2_,eq g^−1^ TiO_2_ h^−1^ after 20 min, compared with 33.1 µmol H_2_,eq g^−1^ TiO_2_ h^−1^ after 45 min for brush deposition. At 120 min, the two coatings retained 56.5 and 42.3% of their respective maximum rates. Integration of the production-rate profiles yielded cumulative specific productions of 151.4 and 39.9 µmol H_2_,eq g^−1^ TiO_2_, respectively, meaning that the value obtained with airbrushing was approximately 3.8 times that obtained with brush deposition. Because these values were normalized by the retained TiO_2_ mass reported in Table S8, the difference cannot be attributed solely to catalyst loading. Instead, the higher mass-normalized response of the airbrushed coating may reflect differences in coating uniformity, catalyst accessibility, irradiation and liquid–solid mass transfer. However, coating porosity, pore architecture and P25 spatial distribution were not independently quantified; therefore, the relative contributions of catalyst accessibility, irradiation and liquid–solid mass transfer to the observed differences cannot be separated from the present data [32,33].

The selected airbrushed P25/PARALOID™ B-72 coating remained active during a 15 h extended-operation test under UVA irradiation using a previously operated reactor (Figure 7d). Its H_2_,eq production rate reached approximately 0.8 µmol H_2_,eq h^−1^ after 4 h and retained approximately 72% and 33% of this maximum after 10 and 15 h, respectively. The 25 mL of 0.1 M solution initially contained 2.5 mmol HCOOH, substantially exceeding the cumulative micromolar H_2_,eq production. Therefore, substantial HCOOH depletion through the gas-forming pathway represented by the H_2_,eq metric is unlikely; however, HCOOH concentration was not analytically monitored, and additional consumption through unquantified reaction pathways cannot be excluded. The observed decline may instead involve coating modification, surface blockage, product accumulation or changes in catalyst accessibility, although their individual contributions could not be distinguished. Reactor-to-reactor reproducibility of the selected airbrushed P25/PARALOID™ B-72 configuration was assessed using two independently prepared reactors (Figure S8a). At 120 min, cumulative specific H_2_,eq production reached 114.3 and 89.7 µmol H_2_,eq g^−1^ TiO_2_ for reactors 1 and 2, respectively, corresponding to a relative percent difference of 24.1%. Short-term reuse was evaluated separately using the same airbrushed reactor (Figure S8b). At a common accumulated incident energy of 5.8 kJ L^−1^, cumulative specific H_2_,eq production was 114.3, 100.0 and 83.2 µmol H_2_,eq g^−1^ TiO_2_ for the initial run, first reuse and second reuse, respectively, corresponding to retentions of 87.5% and 72.8% relative to the initial run. The results therefore show moderate reactor-to-reactor variability and a progressive decrease in photocatalytic response upon reuse, while maintaining measurable activity after two reuse cycles. These experiments demonstrate short-term reusability, whereas the separate 15 h experiment (Figure 7d) provides an assessment of extended operation. Further independent reuse cycles and longer operating periods would be required to assess long-term durability.

Based on these results, the formulation containing 15 wt.% PARALOID™ B-72 and 1200 mg TiO_2_ L^−1^ applied by airbrushing provided the most favorable post-print coating and was retained for the subsequent overall comparison of catalyst-deployment strategies. The operating and coating conditions investigated in this study were evaluated sequentially using a one-factor-at-a-time screening strategy. Therefore, the selected conditions represent the most favorable responses within the experimental ranges investigated and should not be interpreted as a global optimum. Interactions among flow rate, TiO_2_ concentration, irradiation conditions, binder content and other experimental variables were not systematically assessed and would require a multivariable experimental design.

### 3.4. Integration of P25 into the Photoreactor Manufacturing Process

To specifically assess the effect of the stage at which P25 was incorporated into the photoreactor, three fabrication-related strategies were subsequently compared using High Clear resin as the common polymeric matrix: post-print airbrushed resin coating, deposition onto the incompletely cured reactor surface (pre-cure integration), and direct printing of P25-loaded resin (Figure 8). Although PARALOID™ B-72 provided the best overall performance among the post-print binders evaluated in Section 3.3, High Clear resin was used as the common polymeric matrix for the comparison of these fabrication-related integration strategies. This approach minimized the influence of binder chemistry and enabled the effect of the TiO_2_ incorporation stage to be assessed more directly. Because these strategies produced different catalyst distributions and nominal TiO_2_ inventories, the resulting reactor-level rates describe the performance of each configuration rather than the intrinsic activity of P25.

For comparison, H_2_,eq production rates were calculated over common intervals of the nominal recirculation-cycle number (N) to account for the different recirculation-cycle numbers at which the catalyst-integration strategies reached their maximum production rates and to enable direct comparison on the same operating-progress basis. Direct printing achieved the highest H_2_,eq production rate, reaching 2.1 µmol H_2_,eq h^−1^ during the 8–10 recirculation-cycle interval. This value was 4.2 and 13.3 times higher than the maxima obtained with the post-print resin coating (0.5 µmol H_2_,eq h^−1^) and pre-cure integration (0.2 µmol H_2_,eq h^−1^), respectively. Both direct printing and post-print coating showed a progressive increase up to 8–10 recirculation cycles, whereas the pre-cure configuration reached its maximum after 2–4 recirculation cycles and subsequently declined.

During recirculation cycles 16–20, direct printing retained 58.3% of its maximum rate, compared with 50.0% for the post-print coating and 20.8% for the pre-cure configuration. The limited performance of the pre-cure configuration is consistent with the low photocatalytic response previously observed for resin-assisted immobilization and may reflect restricted catalyst accessibility or partial shielding by the cured polymer matrix. Direct printing demonstrated that a P25-containing photoreactor could be fabricated directly from photocatalyst-loaded resin [34,39], although comparison with the other catalyst-deployment strategies requires normalization by the effective reactor volume, as addressed in Section 3.5.

### 3.5. Overall Comparison of Catalyst Deployment Strategies

The catalyst-deployment strategies modified not only the way in which P25 was incorporated into the reactor but also the effective liquid volume available within the assembled reactor. For the immobilized and fabrication-integrated configurations, the deposited or integrated material reduced the available channel height relative to the nominal printed geometry, consequently reducing the liquid volume contained within the irradiated region. For the suspended-P25 configuration, the effective liquid volume of the assembled uncoated reactor was experimentally determined and used directly for volumetric normalization, without converting this volume into an effective channel height. This reduction also shortened the liquid-layer thickness and, consequently, the nominal optical path across the flowing liquid. Therefore, the selected configurations were compared using apparent volumetric productivity calculated from their configuration-specific effective reactor volumes (Figure 9 and Table 1). The TiO_2_ mass basis also differed among configurations, and for direct printing the reported value represents the nominal TiO_2_ inventory incorporated into the polymer matrix, not the fraction effectively exposed to the liquid phase and irradiation. Therefore, apparent volumetric productivity should be interpreted as a reactor-level metric rather than as a measure of intrinsic P25 activity or catalyst-utilization efficiency.

As shown in Table 1, the selected catalyst-deployment strategies resulted in substantially different effective liquid volumes. The validated configuration-specific effective reactor volumes were therefore used to normalize the corresponding H_2_,eq production rates and calculate the apparent volumetric productivities. The complete geometrical and radiometric descriptors are provided in Table S6. These effective liquid volumes were used exclusively for experimental reactor-level productivity normalization and were not used to define the CFD domains. The preliminary CFD model retained the nominal CAD channel geometry, whereas the additional configuration-specific simulations at 4 mL min^−1^ used the representative channel heights reported in Table 1 to assess the hydrodynamic effect of channel constriction. The irradiance ratio was used exclusively to contextualize radiation transmission through each reactor assembly and does not represent photon absorption or photocatalytic conversion efficiency.

When the selected configurations were compared on a volumetric basis, direct printing produced the highest maximum productivity, 821.5 µmol H_2_,eq L_reactor_^−1^ h^−1^ (Figure 9a), corresponding to 1.67 and 2.14 times the maximum productivities obtained with suspended P25 and the selected airbrushed P25/PARALOID™ B-72 coating, respectively. Suspended P25 reached a maximum apparent volumetric productivity of 490.9 µmol H_2_,eq Lreactor^−1^ h^−1^, whereas the airbrushed P25/PARALOID™ B-72 coating reached 384.5 µmol H_2_,eq Lreactor^−1^ h^−1^, corresponding to a 21.7% lower maximum productivity than the suspended configuration. Pre-cure integration showed substantially lower performance (42.2 µmol H_2_,_eq_ L_reactor_^−1^ h^−1^). The higher apparent volumetric productivity of the directly printed reactor reflects the combined effect of its H_2_,eq production rate and its smaller effective reactor volume. It should therefore be interpreted as an architecture-level performance metric rather than as evidence of higher intrinsic photocatalytic activity. The reduced channel height may also favor light delivery across the liquid layer by shortening the optical path, although this effect cannot be separated from differences in catalyst distribution, polymer coverage and surface accessibility.

Productivity retention $\left(R_{P}\right)$ calculated as described in the Supplementary Materials (Equation (S19)), was defined as the ratio between the mean apparent volumetric productivity during the post-maximum operating interval and the maximum apparent volumetric productivity of each configuration. Figure 9b therefore compares the maximum reactor-level response with the mean productivity sustained during the subsequent post-maximum operating interval. Suspended P25 showed the highest retention, with a mean post-maximum productivity corresponding to 81.1% of its maximum value. The airbrushed P25/PARALOID™ B-72 coating retained 47.8%, whereas substantially lower retention values were obtained for the directly printed P25/resin reactor (12.9%) and the pre-cure P25/resin configuration (approximately 0%). Thus, although direct printing provided the highest maximum apparent volumetric productivity, this response decreased substantially during subsequent operation. In contrast, suspended P25 showed the smallest relative decrease after reaching its maximum, while the airbrushed coating exhibited an intermediate post-maximum response. Because the configuration-specific maxima occurred at different operating times, the duration of the post-maximum averaging interval differed among configurations. The normalized profiles in Figure 9c were therefore used to complement $R_{P}$ by comparing the relative post-maximum evolution of the selected configurations.

The normalized post-maximum profiles in Figure 9c were derived from the experimental production-rate curves provided in Figure S7. After aligning each profile at its configuration-specific maximum, the previously used airbrushed reactor retained approximately 82% of its maximum productivity after 180 min, compared with approximately 67% for suspended P25. The non-normalized profiles also confirmed that the airbrushed coating remained photocatalytically active for up to 15 h. However, because the two experiments involved different catalyst deployments, absolute rates and operating histories, this comparison describes relative post-maximum stability and should not be interpreted as evidence of intrinsic kinetic superiority.

Direct comparison with previously reported immobilized photocatalytic systems remains difficult because catalyst composition, sacrificial donor, irradiation conditions, reactor geometry and normalization basis vary considerably among studies. Previous studies have demonstrated photocatalytic H_2_ evolution using immobilized TiO_2_-containing polymer films and pollutant degradation using stereolithography-printed P25/resin architectures [34,39]. These studies provide relevant precedents for polymer-supported and fabrication-integrated TiO_2_ systems, although direct performance comparison with the present reactor is not meaningful because catalyst composition, reaction medium, irradiation conditions, reactor geometry and normalization basis differ substantially. Accordingly, the higher maximum reactor-level productivity obtained by direct printing should not be interpreted as greater intrinsic P25 utilization because the nominal catalyst inventory and exposed catalytic fraction differed among configurations (Table S8). In addition, P25 dispersion within the resin and possible sedimentation during printing were not quantified; therefore, the nominally incorporated TiO_2_ mass does not imply a homogeneous spatial distribution or uniform photocatalyst accessibility within the printed reactor.

Taken together, direct printing provided the highest transient reactor-level productivity, whereas suspended P25 showed the greatest short-term productivity retention. Among the immobilized configurations, the airbrushed P25/PARALOID™ B-72 coating combined comparatively high productivity with a more sustained response and demonstrated short-term reuse. For applications prioritizing sustained operation, this trade-off favors the airbrushed coating $\left(R_{P}=47.8\%\right)$ over the directly printed configuration $\left(R_{P}=11.9\%\right)$, despite the higher transient productivity of the latter. Nevertheless, direct printing of TiO_2_-loaded resin remains a promising fabrication-integrated strategy, if catalyst exposure and sustained accessibility within the polymer matrix can be further improved.

### 3.6. Post-Mortem Structural and Surface Characterization

Post-mortem XRD and SEM–EDS analyses were used to examine the structural and surface characteristics of the reactors in relation to the photocatalytic responses observed in Figure 7, Figure 8 and Figure 9. The corresponding XRD patterns are shown in Figure 10, whereas the post-mortem morphology and Ti distribution are presented in Figure 11. The configurations examined were the uncoated reactor operated with suspended P25, the airbrushed P25/PARALOID™ B-72 coating, the pre-cure P25/resin integration layer and the directly printed P25/resin reactor.

The XRD patterns were dominated by the broad diffuse contribution of the polymeric matrix (Figure 10). The principal P25 reflections—anatase (101) at approximately 25.3° and rutile (110) at approximately 27.4°—could not be resolved unambiguously above this background. Weak features at approximately 30–31° and 44° were not assigned because the additional reflections required for reliable phase identification were absent. Accordingly, quantitative phase fractions and crystallite sizes were not determined [15]. The absence of clearly resolved P25 reflections should not be interpreted as catalyst loss or phase transformation, but rather to the weak TiO_2_ diffraction signal relative to the dominant polymeric background. SEM–EDS revealed clear differences among the catalyst-deployment strategies (Figure 11), with the local elemental quantification summarized in Table S7 and representative EDS spectra provided in Figure S10. Additional SEM and Ti Kα elemental maps acquired from a second independent region of each specimen are provided in Figure S11. Because SEM–EDS probes selected microscopic regions, the elemental compositions reported in Table S7 are local and semiquantitative and should not be interpreted as total reactor composition or as quantitatively representative surface Ti concentrations.

The uncoated reactor operated with suspended P25 showed the characteristic striated surface associated with additive manufacturing, together with particulate deposits and localized Ti-rich regions. Because this reactor did not initially contain immobilized TiO_2_, the detected Ti signal is consistent with post-operational deposition of suspended P25 particles on the channel walls. This accumulation may have contributed to the progressive decline in photocatalytic response, although it cannot be established as the sole underlying mechanism.

The airbrushed P25/PARALOID™ B-72 coating exhibited a comparatively continuous morphology and spatially extended Ti distribution, consistent with its favorable balance between productivity and stability. By contrast, the pre-cure surface contained more visible aggregates and locally Ti-rich regions. Local EDS analysis of the selected field showed a high Ti contribution for the pre-cure surface (69.57 wt.%), whereas a lower local value was obtained for the airbrushed coating (21.99 wt.%; Table S7). These values should be interpreted as semiquantitative local measurements rather than as representative bulk Ti contents. Importantly, the higher local Ti signal observed for the pre-cure surface did not translate into higher photocatalytic performance: the pre-cure reactor reached only 42.19 µmol H_2_,eq L_reactor_^−1^ h^−1^ and decreased to 8.79 µmol H_2_,eq L_reactor_^−1^ h^−1^ at 120 min. Therefore, a high local Ti signal cannot be directly equated with a larger illuminated and reactant-accessible photocatalytic surface [34].

The directly printed reactor presented a compact, polymer-rich morphology with few distinguishable particles. Ti was not included in the normalized quantitative EDS result for the selected field reported in Table S7, although sparse Ti Kα signals were observed in the corresponding elemental map (Figure 11d). Sparse Ti signals were also observed in the second independently mapped region (Figure S11d). Therefore, the absence of a quantified Ti value in Table S7 should not be interpreted as absence of Ti from the printed material. Rather, the mapping results are consistent with a heterogeneous and spatially limited Ti distribution at the analyzed surface. Despite the limited local Ti signal, direct printing provided the highest maximum apparent volumetric productivity (821.5 µmol H_2_,eq L_reactor_^−1^ h^−1^), indicating that local EDS-derived Ti content alone does not describe reactor-level photocatalytic performance. The observed response likely reflects the combined effects of catalyst distribution, polymer coverage, aggregation and liquid-accessible irradiated area.

## 4. Conclusions

A transparent polymeric flat-panel photoreactor was manufactured by vat photopolymerization and functionalized with P25 TiO_2_ through post-print coating, pre-cure integration and direct printing. CFD predicted laminar flow throughout the investigated range (Re = 7.4–36.9), with velocity redistribution and localized increases in wall shear stress at the serpentine bends. The operating flow rate was subsequently selected based on the photocatalytic experiments, with 4 mL min^−1^ providing the reference condition for the immobilized configurations.

Binder formulation and deposition method markedly affected the photocatalytic response of the immobilized P25. The selected airbrushed coating containing 15 wt.% PARALOID™ B-72 on a TiO_2_-mass basis and 1200 mg TiO_2_ L^−1^ of coating formulation combined comparatively high productivity with a sustained photocatalytic response and demonstrated short-term reuse. Direct printing confirmed the feasibility of fabricating a P25-containing reactor directly from photocatalyst-loaded resin and provided the highest maximum apparent volumetric productivity, 821.5 µmol H_2_,eq Lreactor^−1^ h^−1^, corresponding to 1.67 and 2.14 times the maximum productivities obtained with suspended P25 and airbrushed P25/PARALOID™ B-72, respectively. However, its productivity retention was limited to 12.9%, showing that high transient volumetric productivity did not translate into sustained performance. Suspended P25 showed the highest short-term post-maximum retention (81.1%), although appreciable run-to-run variability was observed. The airbrushed coating combined sustained performance with demonstrated short-term reuse while avoiding the catalyst-separation step required for suspended nanoparticles. Although Ti remained detectable on the airbrushed surface after operation, the post-mortem SEM–EDS analysis is local and semiquantitative and cannot be used to quantify catalyst loss or Ti release during operation.

Post-mortem SEM–EDS observations were consistent with a combined influence of TiO_2_ distribution, aggregation, polymer coverage and liquid-accessible irradiated surface on reactor performance, while also showing that local EDS-derived Ti content alone was not representative of photocatalytic activity. XRD phase identification was limited by the dominant polymeric contribution and the weak diffraction signal of the supported P25.

Overall, airbrush immobilization provided the most favorable combination of reactor-level productivity, sustained response and demonstrated short-term reuse among the immobilized surface-coating strategies, whereas direct printing showed the greatest potential for integrating P25 directly into the reactor manufacturing process. Accordingly, the quantitative comparisons reported in this study are restricted to H_2_,eq-based reactor performance; direct H_2_ production and gas selectivity cannot be established without compositional analysis of the evolved gas. Future work should therefore address direct gas-composition analysis, the use of alternative sacrificial electron donors to minimize interference from carbon-containing gaseous products, the development of new photoreactor geometries, and the integration of photocatalysts with improved activity under visible-light irradiation. Long-term catalyst retention and reactor durability should also be further assessed.

## Figures and Tables

**Figure 1:**
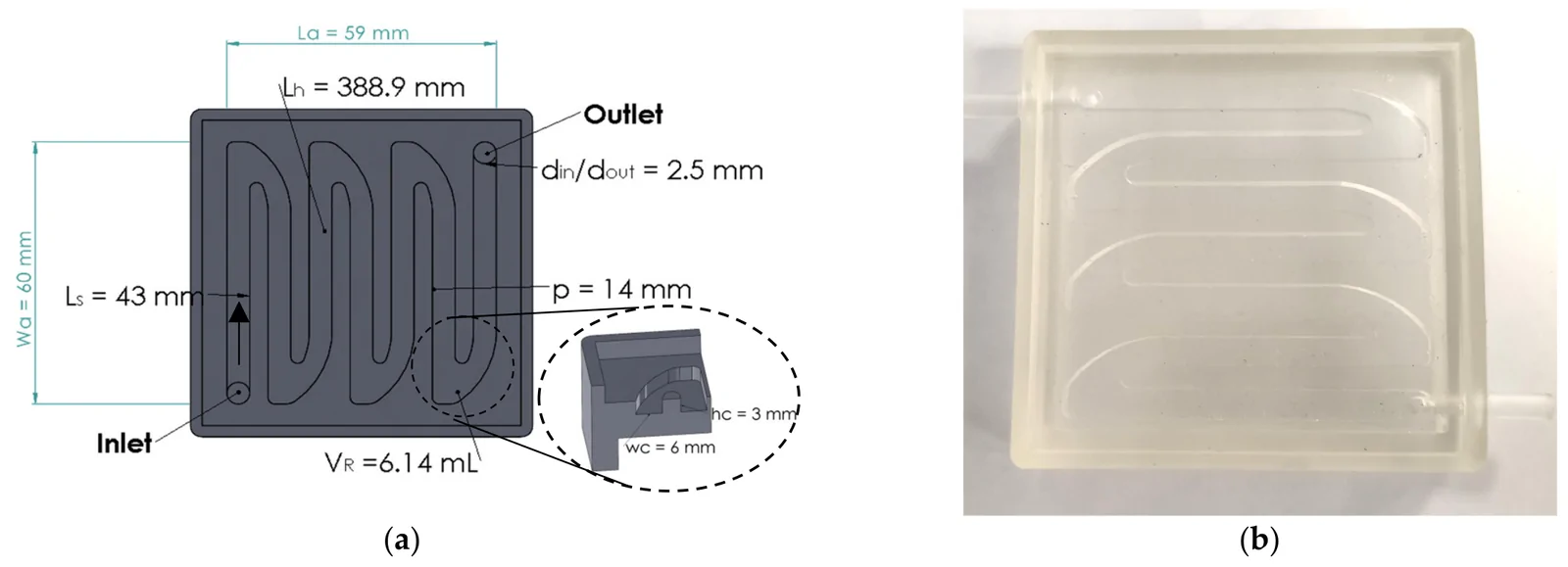
Design and fabrication of the 3D-printed serpentine photoreactor: (**a**) CAD geometry and channel cross-section with the principal dimensions; and (**b**) photograph of the assembled polymeric reactor.

**Figure 2:**
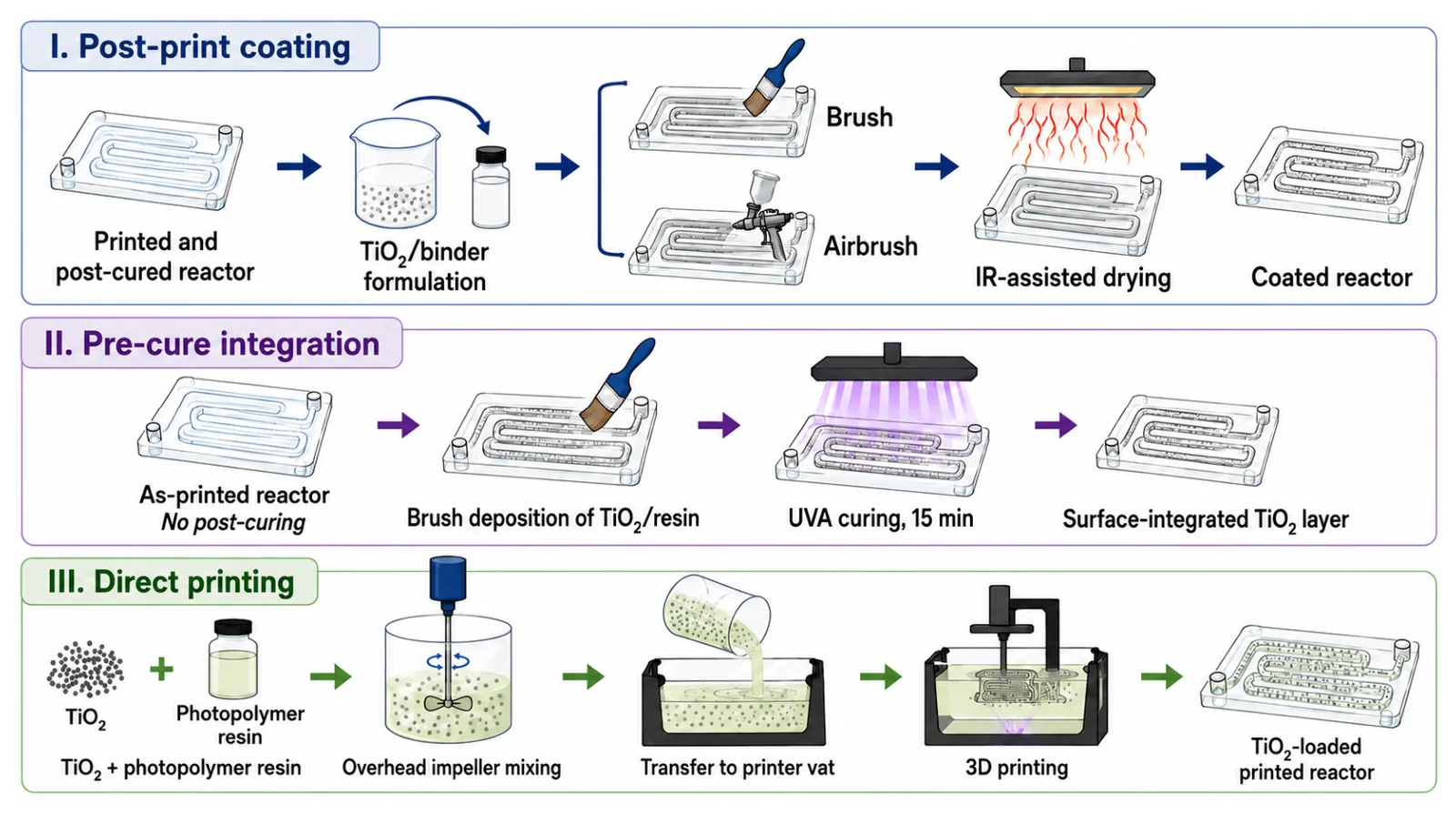
TiO_2_ incorporation strategies evaluated in the polymeric photoreactor: binder-assisted post-print coating, pre-cure integration and direct printing of TiO_2_-loaded resin.

**Figure 3:**
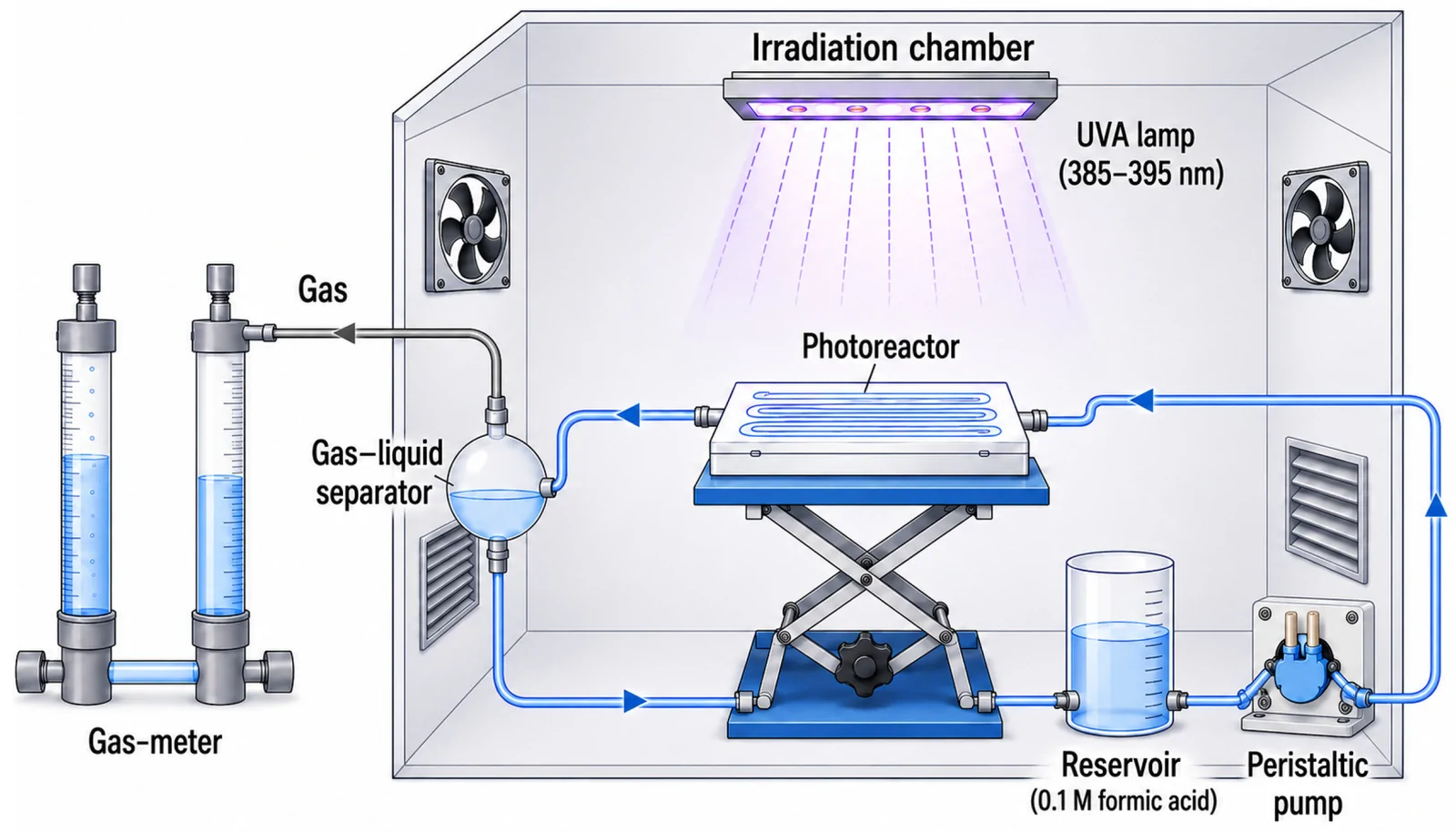
Schematic representation of the recirculating photocatalytic setup.

**Figure 4:**
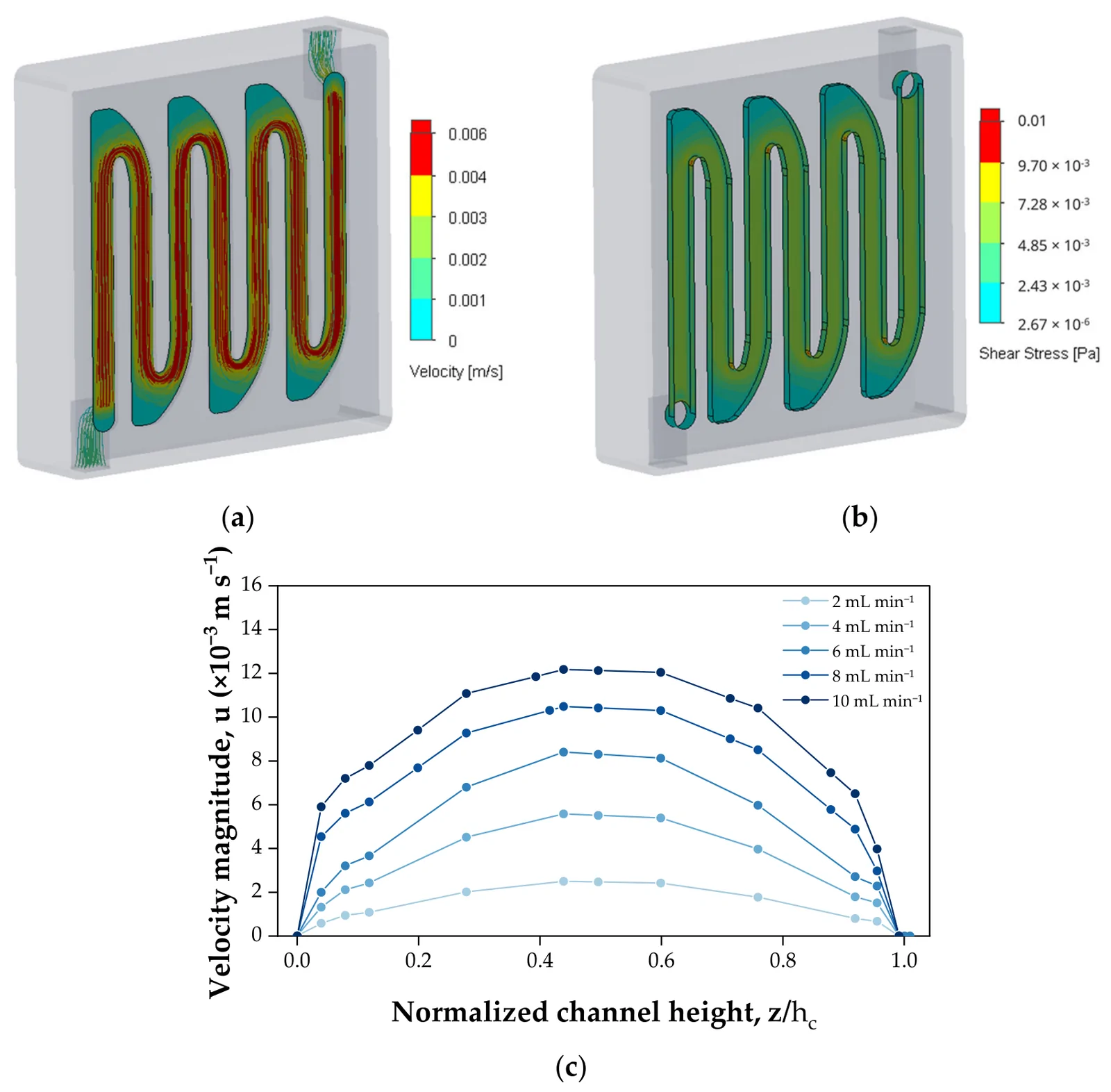
Hydrodynamic assessment of the 3D-printed serpentine photoreactor. (**a**) Velocity-magnitude distribution and flow trajectories at 4 mL min^−1^. (**b**) Wall shear stress distribution over the channel surface at the same flow rate. (**c**) Local velocity-magnitude profiles across the normalized channel height at flow rates of 2–10 mL min^−1^, extracted immediately upstream of a representative 180° bend.

**Figure 5:**
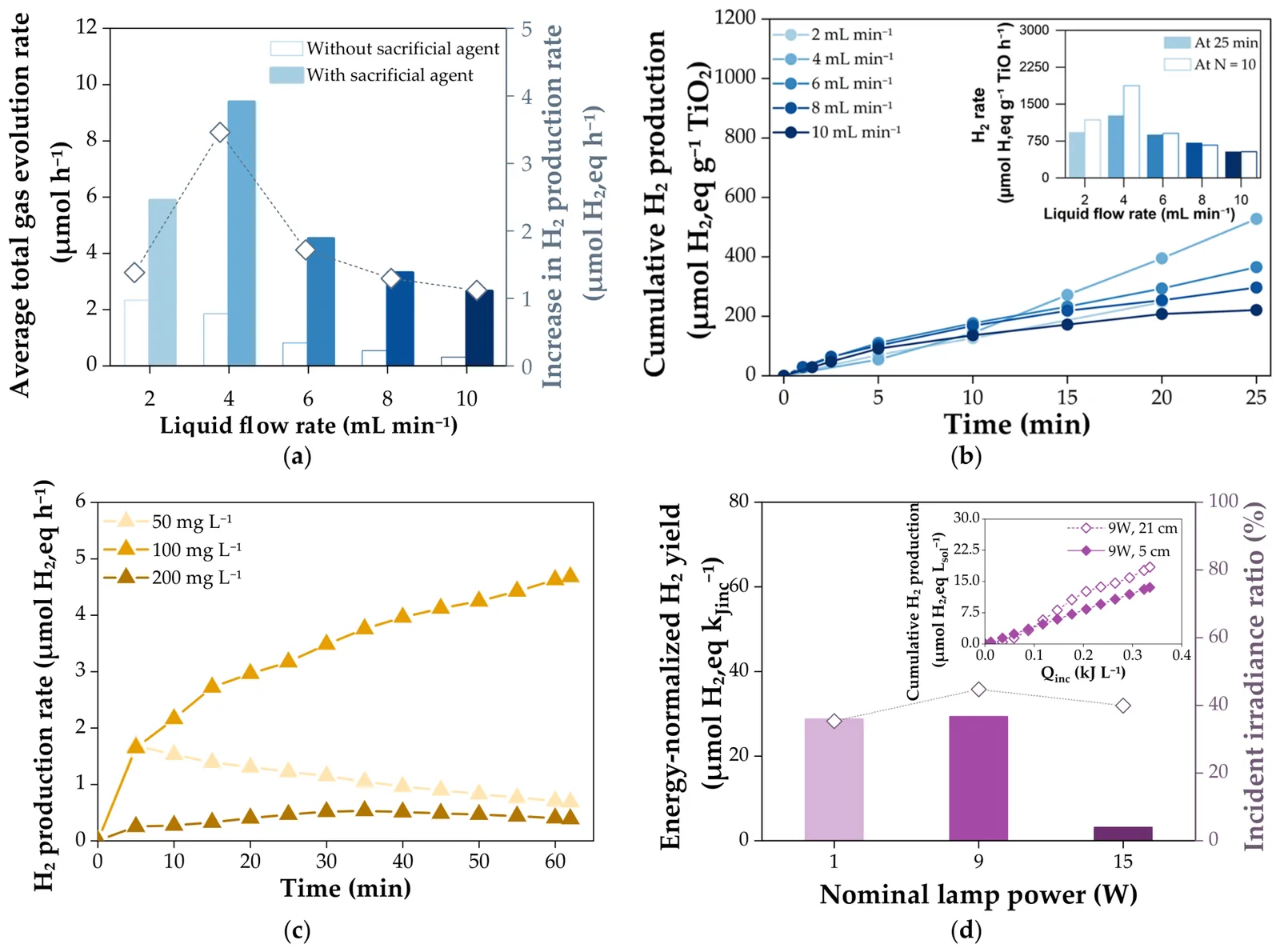
Operating-condition screening using suspended P25: (**a**) total gas evolution obtained with suspended P25 in water and in 0.1 M formic acid solution, with blue shades from light to dark corresponding to liquid flow rates of 2, 4, 6, 8 and 10 mL min^−1^, respectively; (**b**) effect of liquid flow rate; (**c**) effect of TiO_2_ concentration; and (**d**) effect of irradiation conditions. Insets show the corresponding comparison at 25 min and N=10 (**b**), and the energy-normalized response at different lamp-to-reactor distances (**d**).

**Figure 6:**
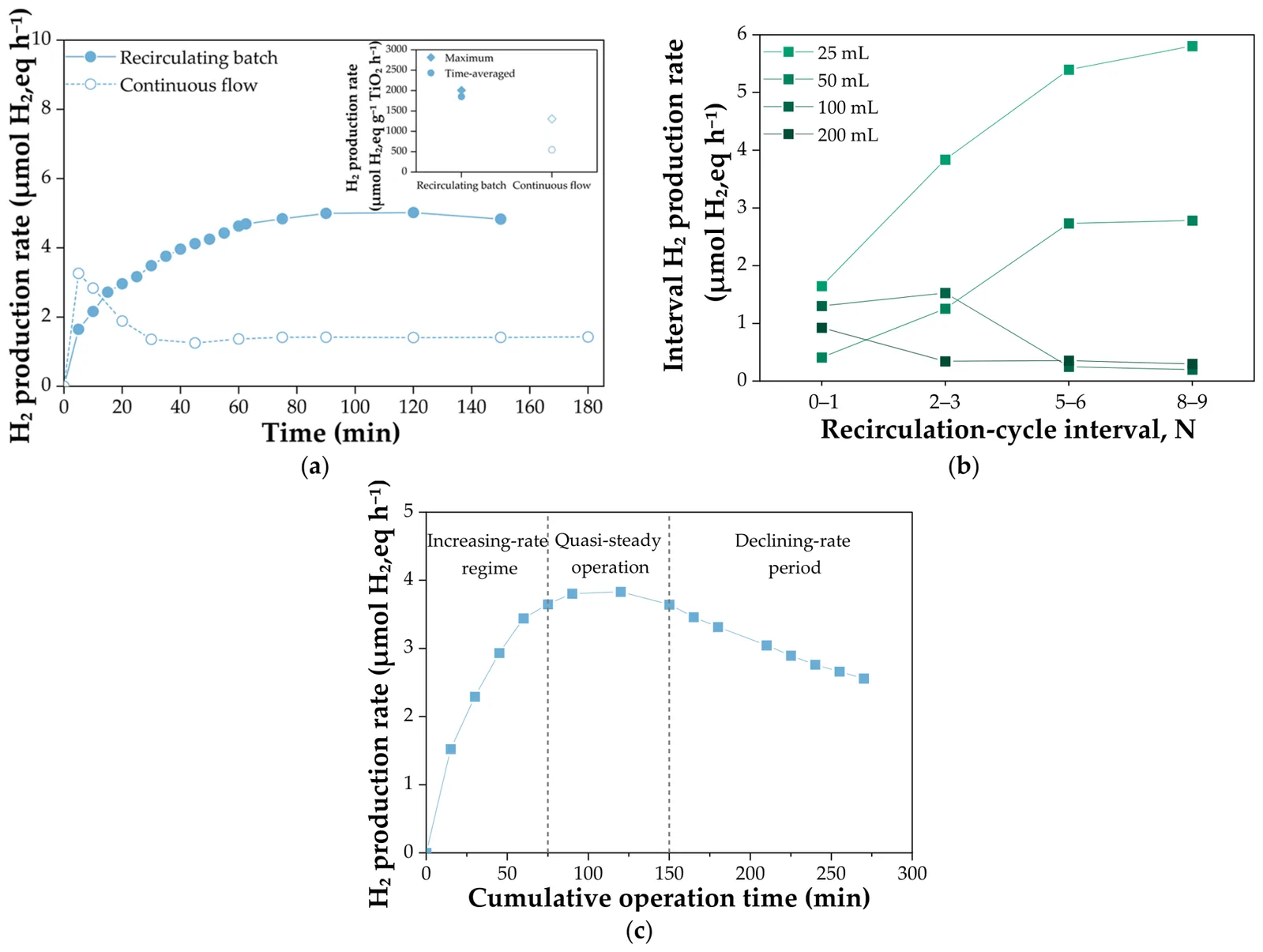
Evaluation of the reactor operating configuration using suspended P25: (**a**) recirculating-batch and continuous flow operation; (**b**) effect of initial liquid volume and recirculation cycles; and (**c**) cumulative operating history of the reused uncoated reactor. The inset in (**a**) compares the maximum and time-averaged specific production rates.

**Figure 7:**
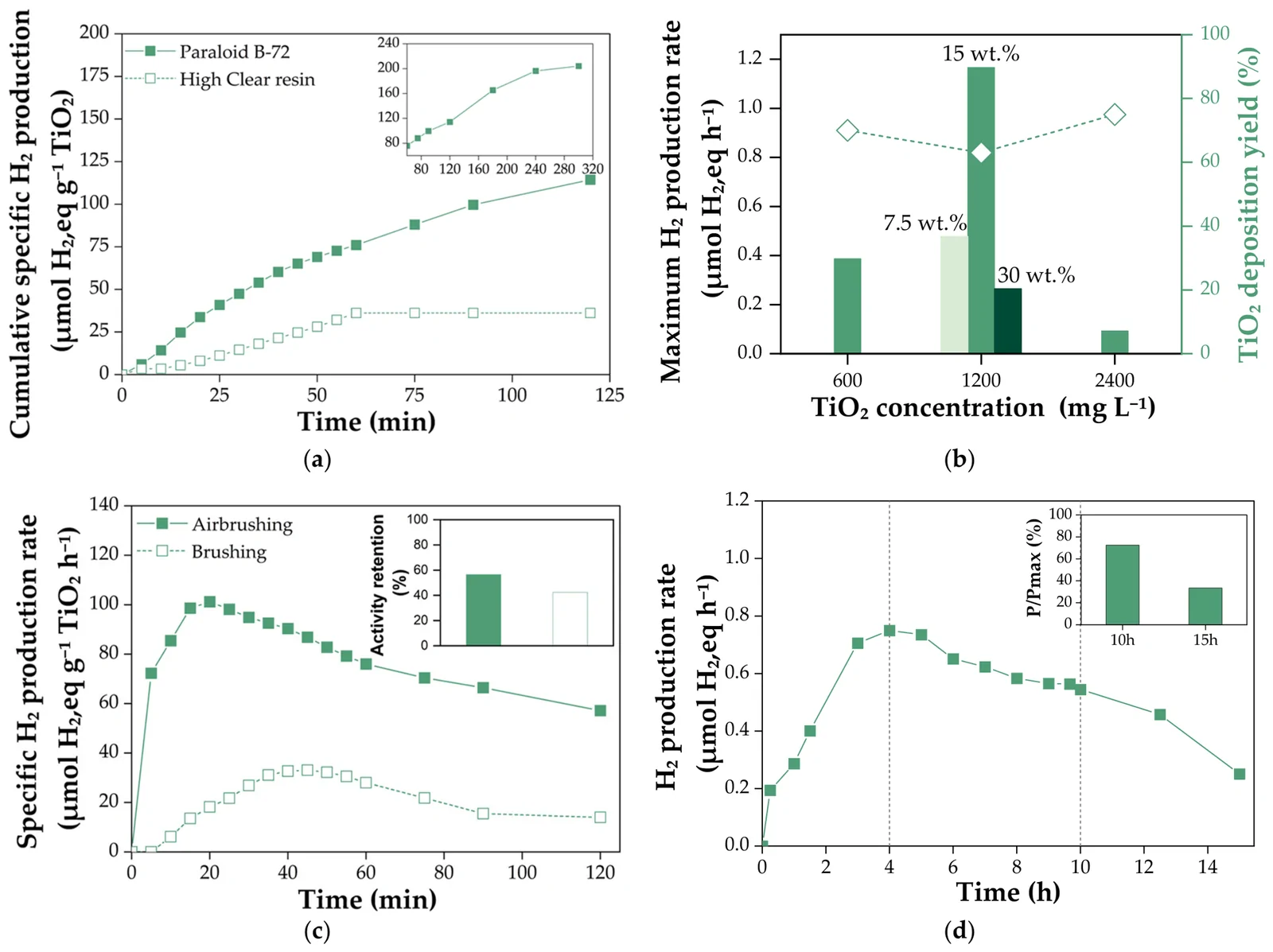
Screening and operational assessment of immobilized P25: (**a**) effect of binder type; (**b**) sequential screening of PARALOID™ B-72 content and P25 concentration (light, medium, and dark green bars correspond to 7.5, 15, and 30 wt.% PARALOID™ B-72, respectively), with TiO_2_ deposition yield shown on the secondary axis; (**c**) comparison of airbrushing and brushing; and (**d**) extended operation of the selected airbrushed P25/PARALOID™ B-72 reactor. Insets show cumulative production (**a**) or productivity-retention metrics (**c**,**d**), as indicated.

**Figure 8:**
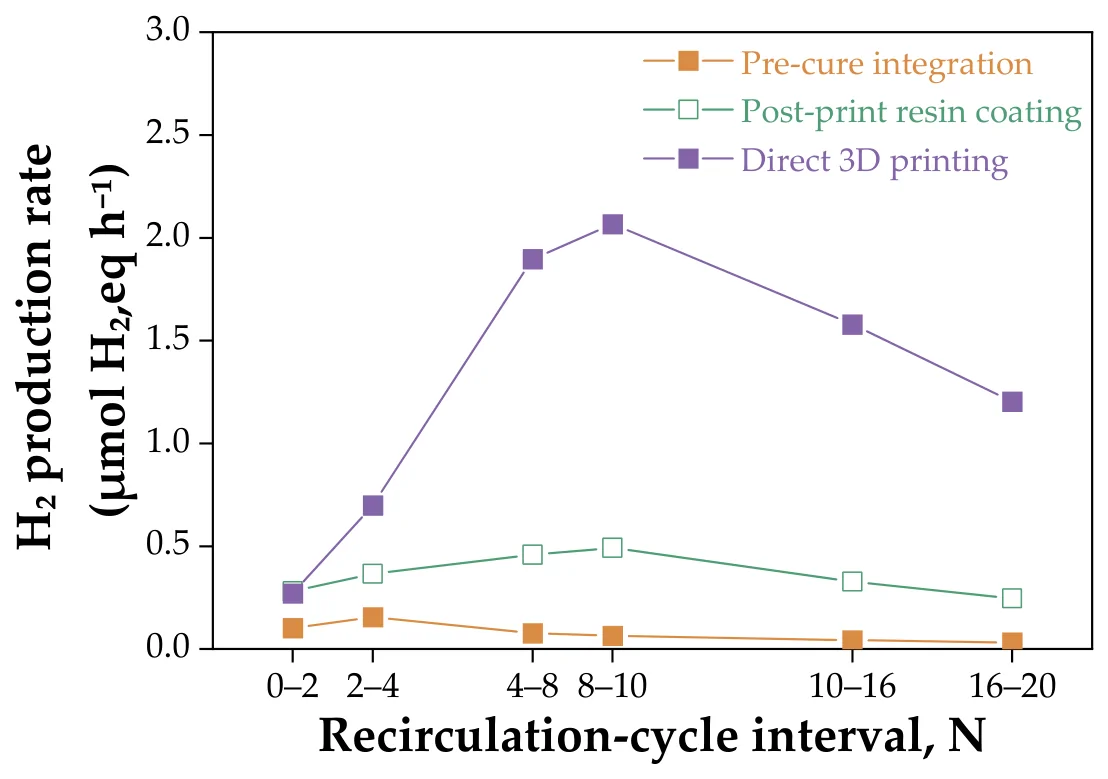
Reactor-level H_2_,eq production rates obtained using post-print airbrushed resin coating, pre-cure integration by brushing and direct printing of P25-loaded resin as a function of cumulative recirculation-cycle interval.

**Figure 9:**
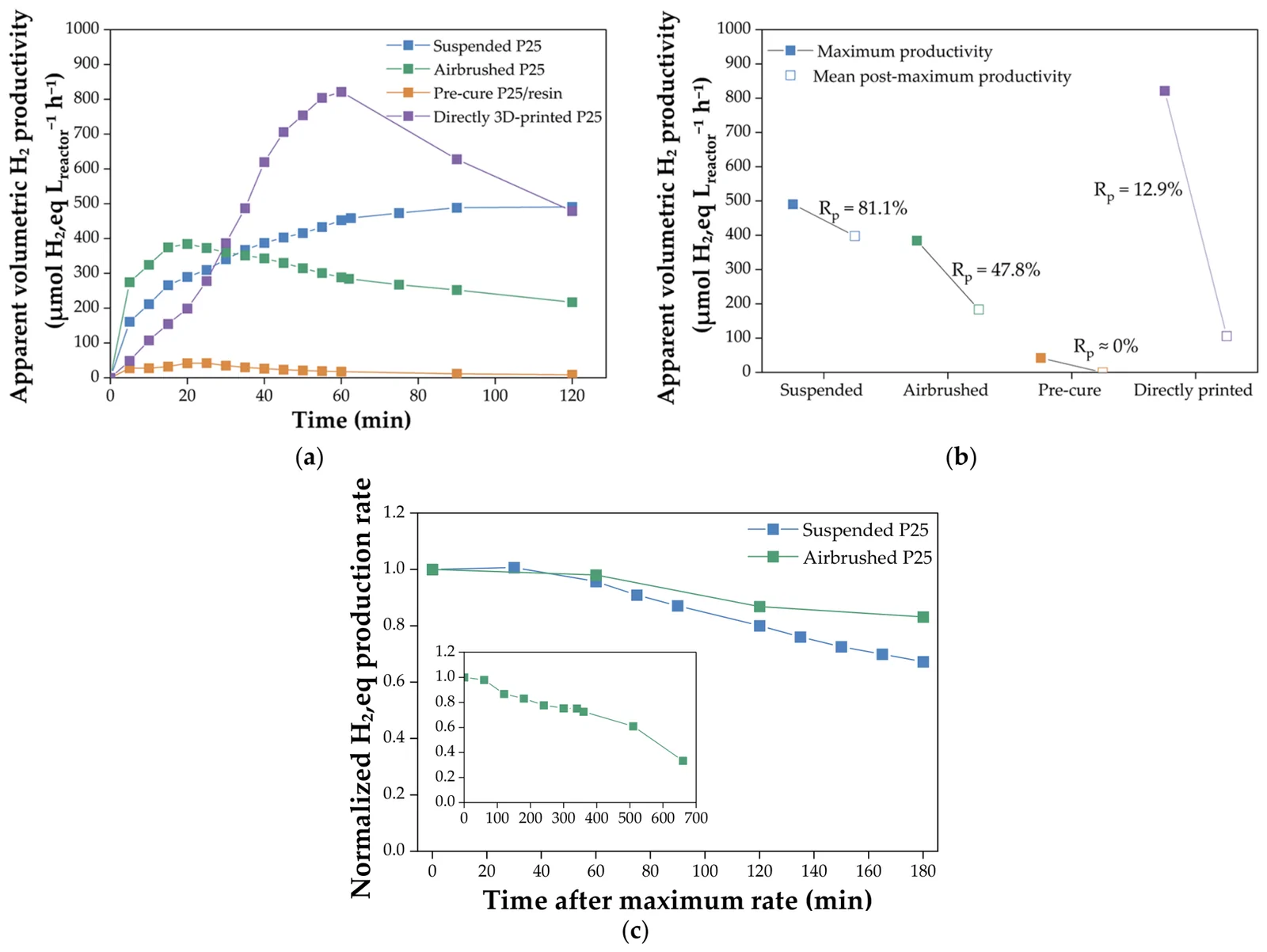
Comparison of the selected photocatalyst configurations: (**a**) apparent volumetric H_2_,_eq_ productivity; (**b**) maximum apparent volumetric productivity and mean post-maximum apparent volumetric productivity for each configuration; and (**c**) normalized post-maximum production profiles of suspended and airbrushed P25/PARALOID™ B-72. R_p_: post-maximum productivity retention. Inset figure in (**c**) shows the extended post-maximum profile of the airbrushed.

**Figure 10:**
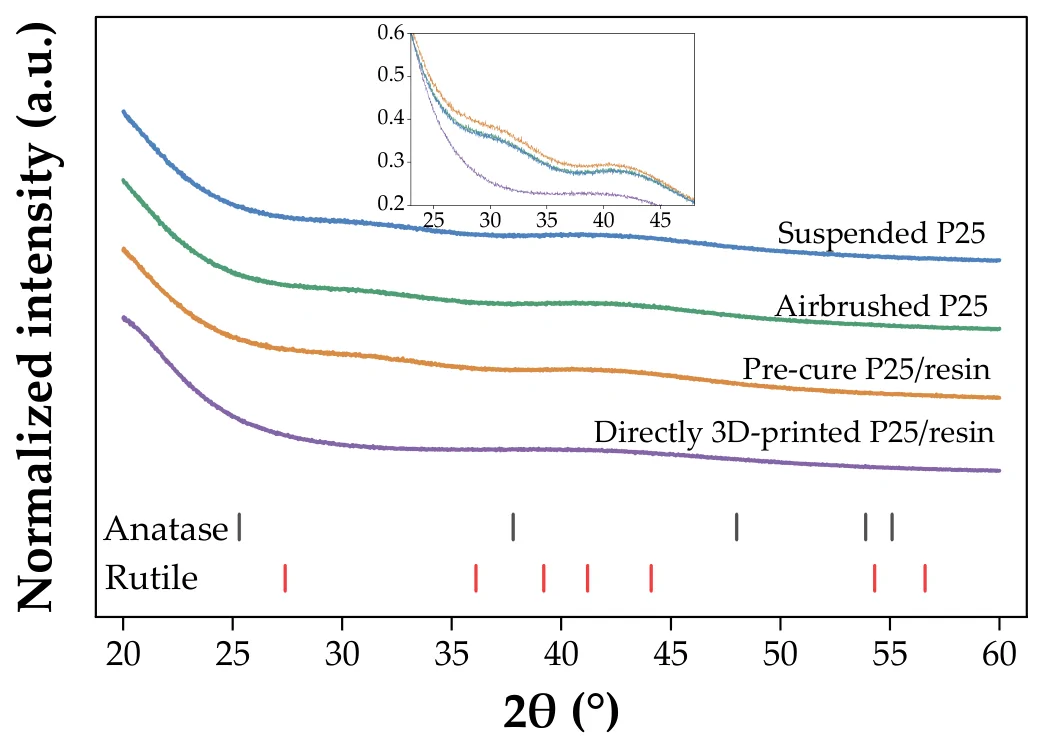
Post-mortem XRD patterns of the uncoated reactor operated with suspended P25 and reactors containing airbrushed P25/PARALOID™ B-72, pre-cure P25/resin integration, and directly printed P25/resin. Colors distinguish the four reactor configurations; gray and red markers denote anatase and rutile, respectively. Patterns were independently normalized and vertically offset for clarity. The inset enlarges the 23–48° region.

**Figure 11:**
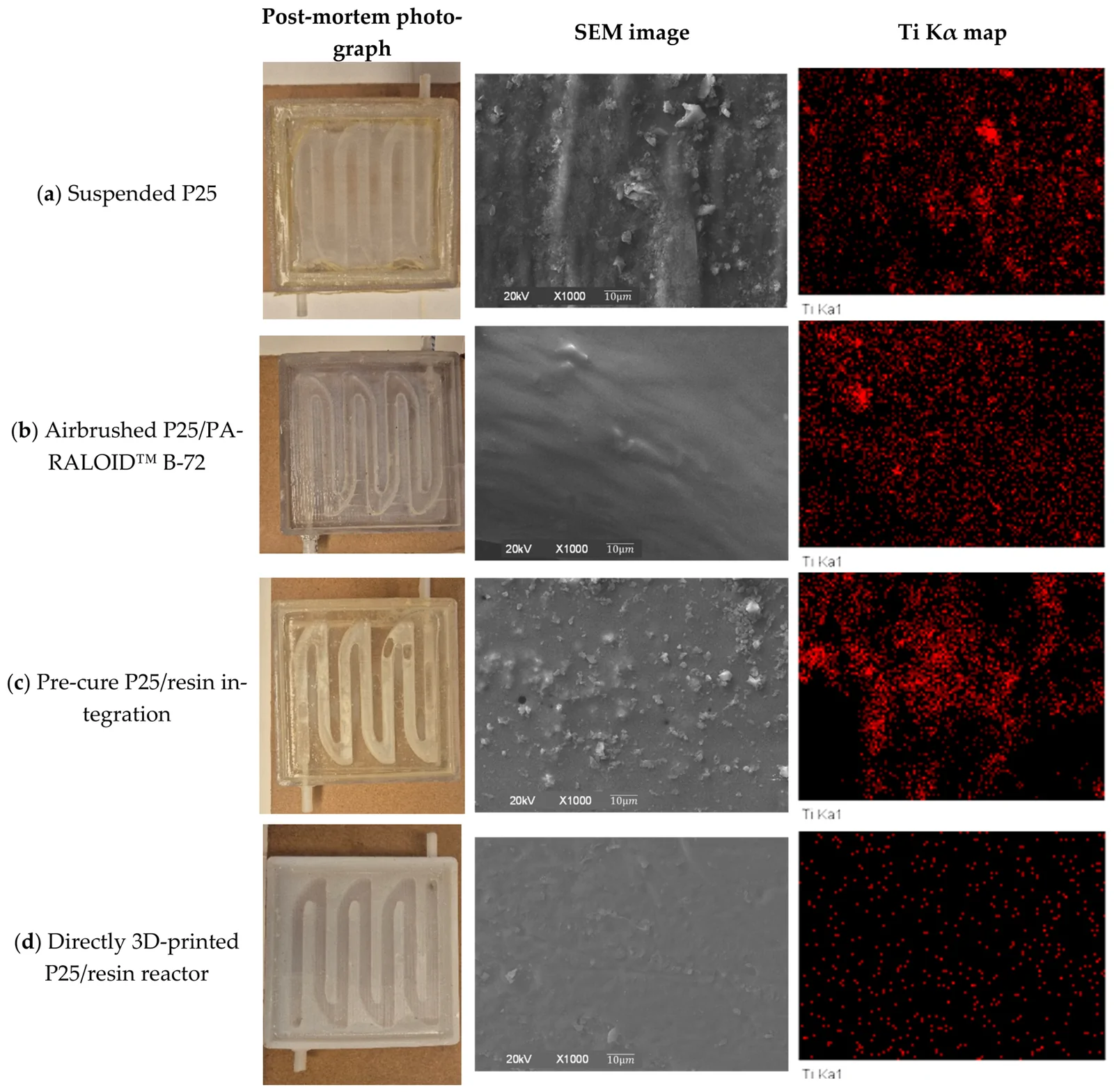
Post-mortem photographs, SEM micrographs and Ti Kα elemental maps of (**a**) the uncoated reactor operated with suspended P25, (**b**) the airbrushed P25/PARALOID™ B-72 coating, (**c**) the pre-cure P25/resin integration, and (**d**) the directly printed P25/resin reactor. SEM micrographs were acquired at 1000× magnification.

**Table 1:** Effective geometry and reactor-level performance metrics of the selected photocatalyst configurations.

Catalyst Configuration	Effective Channel Height h_eff_ (mm) *	Effective Reactor Volume V_R,eff_ (mL) **	Maximum Apparent Volumetric Productivity P_V,max_ (µmol H_2_,eq L_reactor_^−1^ h^−1^)	t_max_ (min)	Productivity Retention R_P_ (%)
Suspended P25	3.00	10.23 ± 0.02	490.9	120	81.1
Airbrushed P25/PARALOID™ B-72	1.68	3.44 ± 0.04	384.5	20	47.8
Pre-cure P25/resin integration	1.80	3.68 ± 0.04	42.2	25	≈0.0
Directly printed P25/resin reactor	1.23	2.51 ± 0.07	821.5	60	12.9

* The preliminary CFD analysis over the 2–10 mL min^−1^ flow-rate range was based on the nominal 3.00 mm channel height defined in the CAD geometry. Additional configuration-specific CFD simulations were performed at 4 mL min^−1^ using the representative channel heights reported in the table. ** V_R,eff_ values are reported as mean ± standard deviation based on the triplicate experimental residence-time measurements (*n* = 3), with the calibrated liquid flow rate treated as fixed.

## Data Availability

The original contributions presented in this study are included in the article/Supplementary Materials. Further inquiries can be directed to the corresponding author.

## Supplementary Materials

The following supporting information can be downloaded at https://www.mdpi.com/article/10.3390/nano16201283/s1: Figure S1: Photographs of the photocatalytic setup and assembled 3D-printed flat-panel photoreactor; Table S1: Main parameters used for additive manufacturing and post-curing of the polymeric photoreactors; Table S2: Mesh-sensitivity analysis performed at a liquid flow rate of 4 mL min^−1^; Table S3: Experimental matrix used in the photocatalytic experiments; Table S4: Control experiments used to assess the background volumetric gas response of the experimental system; Supplementary Note S1: Calculation procedures; Table S5: Theoretical hydrodynamic parameters of the uncoated 6 × 3 mm serpentine channel; Figure S2: CFD-predicted velocity-magnitude distributions in the uncoated serpentine channel at liquid flow rates of 2–10 mL min^−1^; Figure S3: CFD-predicted wall-shear-stress distributions in the uncoated serpentine channel at liquid flow rates of 2–10 mL min^−1^; Figure S4: CFD-predicted velocity-magnitude distributions for the selected reactor configurations at 4 mL min^−1^; Figure S5: CFD-predicted wall-shear-stress distributions for the selected reactor configurations at 4 mL min^−1^; Figure S6: Time-resolved cumulative H_2_,eq production obtained with suspended P25 at liquid flow rates of 2–10 mL min^−1^; Figure S7: Short-term repeatability of the photocatalytic experiments conducted at 4, 8 and 10 mL min^−1^; Figure S8: Assessment of the selected airbrushed P25/PARALOID™ B-72 configuration: reactor-to-reactor reproducibility and sequential reuse; Table S6: Effective geometrical parameters and apparent irradiance transmission ratios of the selected reactor configurations; Figure S9: Experimental H_2_,eq production-rate profiles of suspended P25 and the previously used airbrushed P25/PARALOID™ B-72 reactor; Table S7: Instrument-reported local SEM–EDS results for the post-mortem reactor surfaces; Figure S10: Representative EDS spectra of the post-mortem reactor surfaces; Figure S11: Additional SEM micrographs and Ti Kα elemental maps acquired from a second independent region of the post-mortem reactor surfaces; Table S8: TiO_2_ inventory and mass-normalization basis of the selected catalyst-deployment configurations.

## Abbreviations

The following abbreviations are used in this manuscript:

CAD	Computer-Aided Design
CFD	Computational Fluid Dynamics
EDS	Energy-Dispersive X-ray Spectroscopy
H_2_,eq	Stoichiometric Hydrogen Equivalent
IR	Infrared
LCD	Liquid Crystal Display
SEM	Scanning Electron Microscopy
T_I_	Apparent Irradiance Transmission Ratio
UVA	Ultraviolet A
XRD	X-ray Diffraction
