# Supplementary material for: Surface-Engineered 3D-Printed Polymeric Flat-Panel Photoreactors with P25 TiO2 Nanoparticles for Photocatalytic Hydrogen Evolution
Source: Nanomaterials (Basel). 2026 Oct 10;16(20):1283. doi: 10.3390/nano16201283 (PMC13707699; doi:10.3390/nano16201283)
Supplement: Supplementary file 1 [file nanomaterials-16-01283-s001.zip › nanomaterials-4593896-supplementary.pdf]

Supplementary materials

Surface-Engineered 3D-Printed Polymeric Flat-Panel Photoreactors with P25 TiO_2 Nanoparticles for Photocatalytic Hydrogen Evolution

María José Torres, Jesús Román, María del Prado Garrido, Mireya Carvela*

Hydrogen Technologies and Energy Transition Department, Association for Research and Technological Development of Industry in Castilla-La Mancha (ITECAM), Ctra. Pedro Muñoz, Km. 1, 13700 Tomelloso, Ciudad Real, Spain.

Correspondence: mireya.carvela@itecam.com

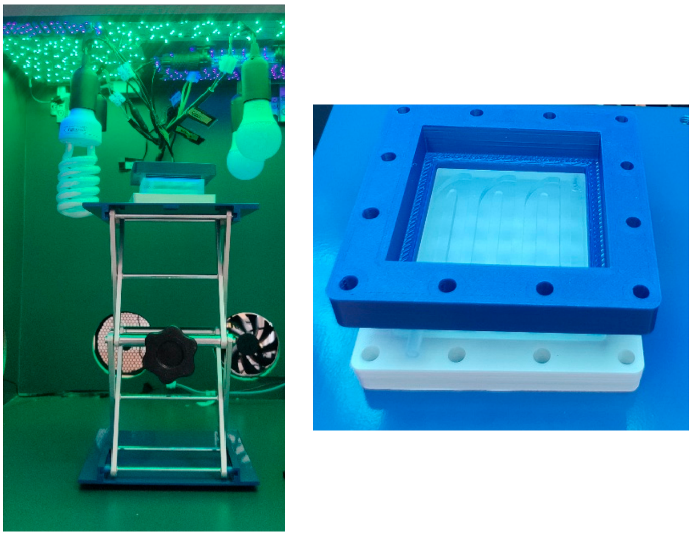

(**a**) (**b**)

Figure S1. Photographs of (**a**) the photocatalytic setup inside the irradiation chamber and (**b**) the assembled 3D-printed flat-panel photoreactor.

Table S1. Main parameters used for additive manufacturing and post-curing of the polymeric photoreactors.

Parameter	Value
Manufacturing technology	LCD-based masked stereolithography
Printer	Anycubic Photon M3 Max
Photopolymer	High Clear resin
Layer thickness (mm)	0.05
Normal exposure time (s)	6
Light-off time (s)	2.5
Bottom exposure time (s)	30
Bottom layers	6
Washing solvent	2-Propanol
Post-curing conditions	Ultraviolet A (UVA), 60 °C, 15 min

Table S2. Mesh-sensitivity analysis performed at a liquid flow rate of 4 mL min^{-1}.

Result-resolution level	Base mesh Nx × Ny × Nz	Fluid cells	Iterations	Global average velocity ($m\ s^{-1}$)	Global maximum velocity ($m\ s^{-1}$)	Average wall shear stress (Pa)	Normalized flow residual ε_Q (%)
5	56 × 56 × 12	112,713	262	0.002	0.0067	0.072	1.32×10^{-3}
6	80 × 80 × 16	311,013	347	0.002	0.0067	0.072	5.52×10^{-4}
7	112 × 112 × 24	855,852	451	0.002	0.0067	0.072	4.56×10^{-4}

*The normalized flow residual was calculated as $\varepsilon_Q = |Q_{net}|/Q_{in} \times 100$, where Q_{net} is the global net volumetric flow rate and Q_{in} is the imposed inlet flow rate. Global velocity values include the inlet and outlet connection regions and were used exclusively for mesh-sensitivity assessment; consequently, they should not be directly compared with the active-channel velocity fields shown in Figure 4 and Figures S2–S3.

Table S3. Experimental matrix used in the photocatalytic experiments.

(A) Suspended-P25 experiments

Experimental series	Evaluated conditions	Operating endpoint
Sacrificial-agent control	Water; 0.1 M HCOOH	-
Flow-rate screening	2, 4, 6, 8 and 10 mL min^{-1}	-
P25-loading screening	50, 100 and 200 mg L^{-1}	-
Lamp-power screening	1, 9 and 15 W	Common incident-energy basis
Lamp-distance assessment	5 and 21 cm; 9 W	$Q_{inc} \leq 0.34$ kJ L^{-1}
Operating-mode comparison	Recirculating batch; continuous flow	Recirculating batch: 10 cycles; continuous flow: 3 h
Liquid-volume screening	25, 50, 100 and 200 mL	-
Reactor reuse	Three consecutive experiments	270 min cumulative

(B) Binder-assisted coatings

Experimental series	Evaluated conditions	Fixed condition / coating
Binder screening	Paraloid B-72; High Clear resin	Airbrush
Binder-content screening	7.5, 15 and 30 (wt. %) Paraloid	Airbrush
TiO_2-concentration screening	600, 1200 and 2400 mg L^{-1}	Airbrush
Deposition-method comparison	Airbrush; brush	15% Paraloid, 1200 mg TiO_2 L^{-1}
Short-term reuse	Two sequential runs	Selected airbrushed coating
Prolonged operation	Previously used reactor; 15 h	Selected airbrushed coating

(C) Manufacturing-integrated configurations

Configuration	TiO_2 incorporation route
Post-print resin coating	TiO_2/resin applied after reactor post-curing
Pre-cure integration	TiO_2/resin applied before final UVA curing
Direct printing	1200 mg L^{-1} P25 dispersed in the printing resin

*Unless otherwise specified, the standard conditions defined above were applied. The nominal number of recirculation cycles was calculated as $N = Q_t/V_0$. Fresh 0.1 M formic-acid solution was used after washing in the reuse experiments.

**Screening experiments were performed as single experimental runs (n = 1). Replicated experiments used for repeatability, reactor-to-reactor reproducibility and sequential reuse are described separately in the main manuscript and corresponding supplementary figure.

Table S4. Control experiments used to assess the background volumetric gas response of the experimental system.

Condition	Control condition	Accumulated apparent gas (µmol gas)
Water + UVA	Without P25 and HCOOH	0.077
0.1 M HCOOH + UVA	Without P25	0.032
P25 + 0.1 M HCOOH, dark	Without UVA irradiation	0.026

* The control experiments were used to assess possible non-photocatalytic contributions to the volumetric gas signal. Because the three conditions do not constitute a common instrumental blank, these values were not subtracted from the photocatalytic datasets.

Supplementary Note S1. Calculation procedures

- **S1.1. Hydrodynamic calculations**

1) The steady-state continuity and momentum equations were solved by assuming an incompressible Newtonian liquid. No-slip and hydraulically smooth conditions were imposed at the channel walls, whereas heat transfer, gravitational effects and free-surface phenomena were neglected. The hydraulic diameter of the rectangular channel was calculated as:

$$D_h = \frac{2w_c h_c}{w_c + h_c} \quad \text{(S1)}$$

where wc and hc are the nominal channel width and height, respectively. For wc = 6 mm and hc = 3 mm, $D_h = 4$ mm. The theoretical cross-section-averaged velocity and Reynolds number were determined from:

$$\bar{u}_{th} = \frac{Q}{w_c h_c} \quad \text{(S2)}$$

$$Re = \frac{\rho \bar{u} D_h}{\mu} \quad \text{(S3)}$$

where and are the density and dynamic viscosity of water, respectively. Reynolds numbers ranged from approximately 7.4 to 36.9 over the investigated flow-rate interval, confirming laminar-flow conditions. No-slip and hydraulically smooth conditions were imposed on the channel walls. Heat transfer was not included in the CFD model. Experimentally, the circulating liquid remained below 30 °C under the irradiation conditions investigated. As a sensitivity check, the Reynolds number was recalculated using the properties of water at 30 °C, giving values of approximately 9.3–46.3 over the investigated flow-rate range, compared with 7.4–36.9 at 293.2 K. Thus, the flow remains clearly within the laminar regime over the experimentally observed temperature range.

The CFD results were therefore interpreted as an isothermal hydrodynamic description, while temperature-dependent variations in pressure drop and wall shear stress were not explicitly considered.

2) Numerical convergence was monitored using the inlet and outlet volumetric flow rates, average and maximum velocities, inlet and outlet static pressures, and average wall shear stress. All final calculations reached the solver convergence criterion, and the relative volumetric-flow imbalance,

$$\varepsilon_{\mathrm{Q}} = \frac{|Q_{\mathrm{in}} + Q_{\mathrm{out}}|}{|Q_{\mathrm{in}}|} x\ 100 \qquad \text{(S4)}$$

remained below 0.002%. Numerical consistency was additionally assessed by comparing the section-averaged CFD velocity with $\bar{\mu}_{\mathrm{th}}$.

3) Velocity fields, flow trajectories, pressure and wall-shear-stress distributions were evaluated throughout the reactor. Transverse axial-velocity profiles were extracted from the same straight-channel section immediately upstream of a bend. The nominal CFD residence time (τ_{CFD}) and pressure drop were calculated as:

$$\tau_{\mathrm{nom}} = \frac{V_{\mathrm{R}}}{Q} \qquad \text{(S5)}$$

$$\Delta P = \bar{P}_{\mathrm{in}} - \bar{P}_{\mathrm{out}} \qquad \text{(S6)}$$

where V_R is the nominal internal reactor volume and $\bar{P}_{\mathrm{in}} - \bar{P}_{\mathrm{out}}$ are the area-averaged static pressures at the inlet and outlet, respectively.

Table S5. Theoretical hydrodynamic parameters of the uncoated 6 × 3 mm serpentine channel.

Flow rate (mL min^{-1})	Mean velocity (m s^{-1})*	Reynolds number	Nominal CFD residence time, τ_{CFD} (s)**	Recirculation cycle time (min)
2	1.85 x 10^{-3}	7.4	184.1	12.50
4	3.70 x 10^{-3}	14.8	92.0	6.25
6	5.56 x 10^{-3}	22.1	61.4	4.17
8	7.41 x 10^{-3}	29.5	46.0	3.13
10	9.26 x 10^{-3}	36.9	36.8	2.50

*Mean velocity was calculated as u = Q/($w_c h_c$) using wc = 6 mm and hc = 3 mm. The Reynolds number was calculated using a hydraulic diameter of 4 mm and the properties of water at 293.2 K. The nominal residence time was calculated as $\tau_r = V_R/Q$, using V_R = 6.135 mL. Recirculation-cycle time corresponds to V_0/Q for an initial liquid volume of 25 mL.

**The nominal CFD residence time was calculated as $\tau_{\mathrm{CFD}} = \frac{V_{\mathrm{R,nom}}}{Q}$, using $V_{\mathrm{R,nom}}$ = 6.14 mL.

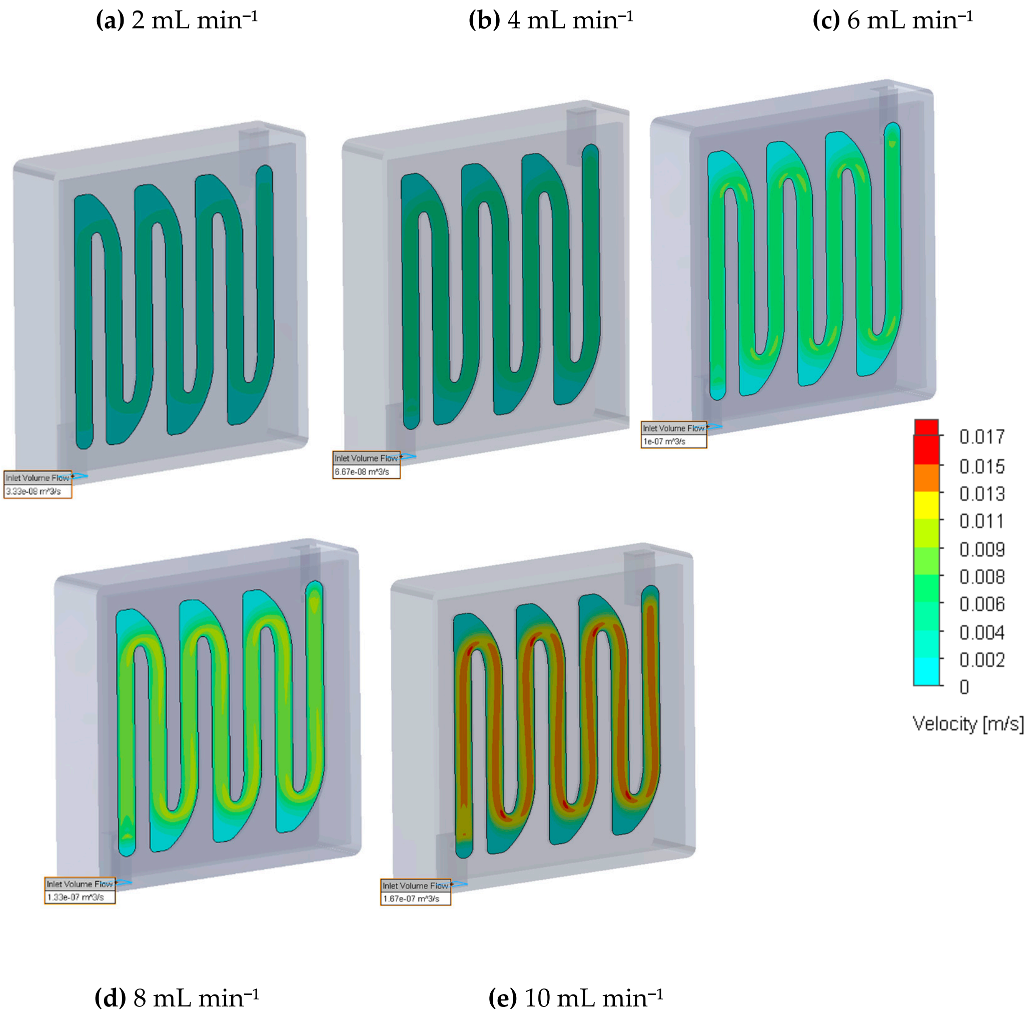

Figure S2. CFD-predicted velocity-magnitude distributions in the uncoated serpentine channel at liquid flow rates of 2–10 mL min^{-1}. CFD-predicted velocity-magnitude distributions in the uncoated 6 mm × 3 mm serpentine channel at liquid flow rates of (**a**) 2, (**b**) 4, (**c**) 6, (**d**) 8 and (**e**) 10 mL min^{-1}. A common color scale is used for all operating conditions.

(a) 2 mL min^{-1} **(b)** 4 mL min^{-1} **(c)** 6 mL min^{-1}

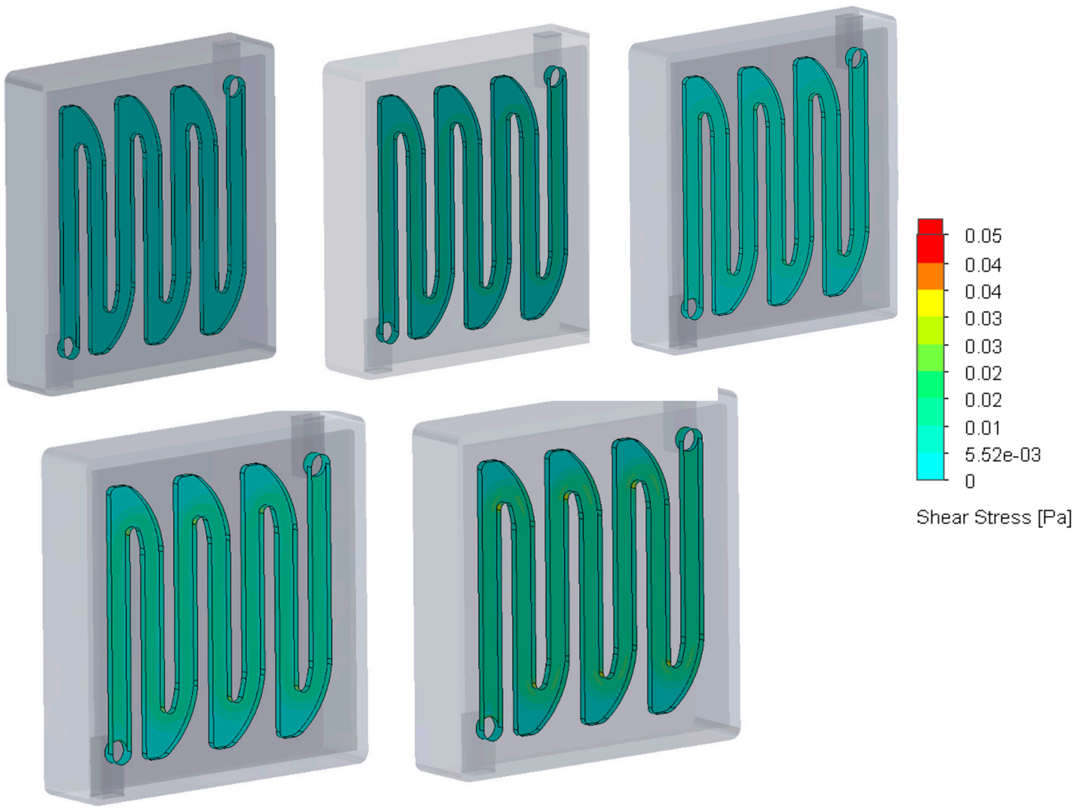

(d) 8 mL min^{-1} **(e)** 10 mL min^{-1}

Figure S3. CFD-predicted wall-shear-stress distributions in the uncoated serpentine channel at liquid flow rates of 2–10 mL min^{-1}. CFD-predicted wall-shear-stress distributions in the uncoated 6 mm × 3 mm serpentine channel at liquid flow rates of (**a**) 2, (**b**) 4, (**c**) 6, (**d**) 8 and (**e**) 10 mL min^{-1}. A common color scale is used for all operating conditions.

To complement the nominal-channel analysis, the influence of the configuration-specific channel constriction was evaluated at 4 mL min^{-1} using the representative channel heights of the selected catalyst-deployment configurations. The resulting velocity-magnitude and wall-shear-stress distributions are shown in Figure S4 and Figure S5.

(**a**) Suspended P25

(**b**) Airbrushed P25/ PARALOID™ B-72

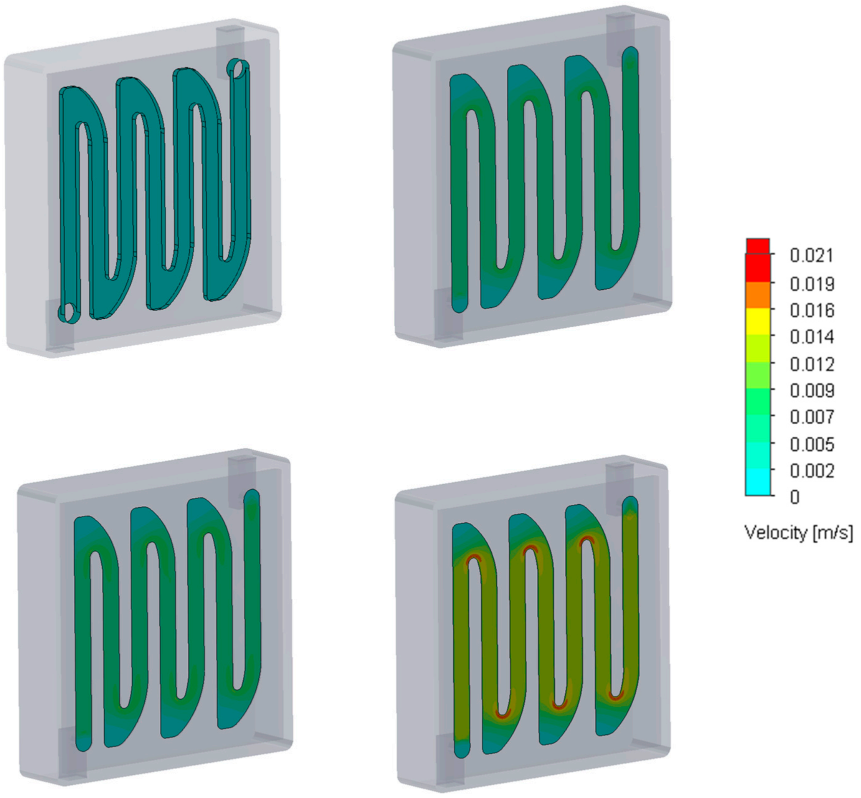

(c) Pre-cure P25/resin integration

(d) Directly 3D-printed TiO_2/resin reactor

Figure S4. CFD-predicted velocity-magnitude distributions for the selected reactor configurations at 4 mL min^{-1}.

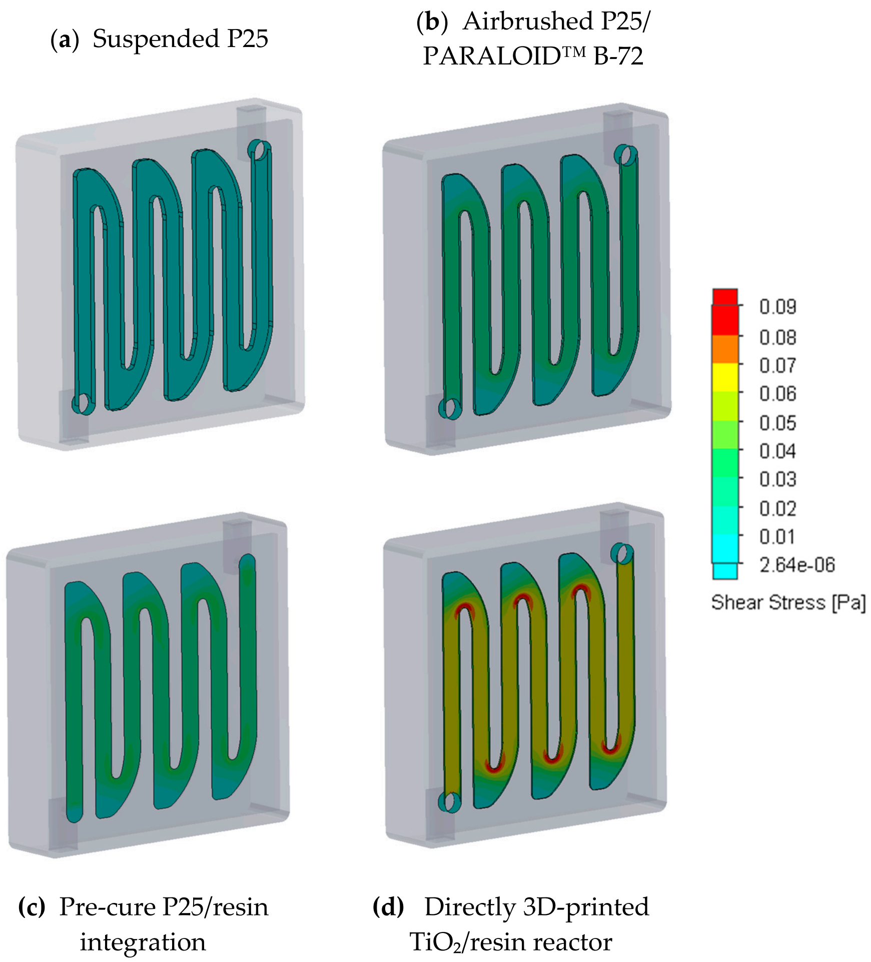

Figure S5. CFD-predicted wall-shear-stress distributions for the selected reactor configurations at 4 mL min^{-1}.

- **S1.2. Photocatalytic performance calculations**

  The conversion of the displaced gas volume into η_{gas}, the calculation of $\eta_{H_{2,eq}}$, the apparent irradiance transmission ratio (T_I) and the nominal number of liquid recirculation cycles (N) were performed according to Equations (6) – (9) of the main manuscript. The additional performance metrics used for data analysis are defined below.

1) The H_2-equivalent (H_2,eq) production rate between two consecutive measurements was calculated from:

$$r_{H_{2,eq,i}} = \frac{\eta_{H_{2,eq}}(t_i) - \eta_{H_{2,eq}}(t_{i-1})}{t_i - t_{i-1}} \tag{S7}$$

where the time interval was expressed in hours. Cumulative mass-specific production and mass-specific production rate were calculated as:

$$N_{H_{2,eq}}(t) = \frac{\eta_{H_{2,eq}}(t)}{m_{TiO_2}} \tag{S8}$$

$$r_{\mathrm{H_2,eq,m}}(t) = \frac{r_{\mathrm{H2,eq}}}{m_{\mathrm{TiO_2}}} \tag{S9}$$

where m_{TiO_2}denotes the catalyst mass dispersed in the liquid, retained within the coating or nominally incorporated into the printed composite, depending on the catalyst-deployment strategy. These parameters are reported as μmol H_2,eq g^{-1} TiO_2 and μmol H_2,eq g^{-1} TiO_2 h^{-1}, respectively.

2) Configuration-specific effective reactor liquid volumes were determined experimentally from the calibrated liquid flow rate and the mean experimental residence time measured in triplicate, according to $V_{\mathrm{R,eff}} = Q_{\mathrm{cal}}\, \tau_{\mathrm{exp}}$. The resulting $V_{R,eff}$ values were 10.23, 3.44, 3.68 and 2.51 mL for suspended P25, airbrushed P25/PARALOID™ B-72, pre-cure P25/resin and directly printed P25/resin, respectively. These values were used for reactor-level volumetric normalization and were treated independently from the nominal 6 mm × 3 mm CAD/CFD geometry ($V_{\mathrm{R,nom}}$ = 6.14 mL). The apparent volumetric H_2,eq productivity was calculated as:

$$P_{\mathrm{V}}(t) = \frac{r_{\mathrm{H2,eq}}}{V_{\mathrm{R,eff}}} \tag{S10}$$

where $r_{H_2,eq}(t)$ is the H_2,eq production rate and $V_{R,\mathrm{eff}}$ is the configuration-specific effective liquid volume of the assembled reactor. P_V(t) is reported as μmol H_2,eq $\mathrm{Lreactor}^{-1}$ h^{-1}. The experimentally determined effective liquid volumes were used only for reactor-level productivity normalization and were not used to define the CFD domains. The primary CFD analysis retained the nominal CAD geometry, whereas the additional configuration-specific simulations at 4 mL min^{-1} used the representative channel heights to evaluate the hydrodynamic effect of channel constriction.

3) The cumulative radiant energy incident on the projected irradiated channel area was calculated as:

$$E_{\mathrm{inc}} = \frac{q''_{\mathrm{inc}} A_{\mathrm{irr}} t}{1000}, \tag{S11}$$

where q''_{inc} is the irradiance measured at the reactor plane in W m^{-2}, A_{irr} is the projected irradiated channel area in m^2 and t is the irradiation time in seconds. The factor 1000 converts joules into kilojoules. The incident energy normalized by the initial liquid volume was calculated as:

$$E_{\mathrm{inc},V} = \frac{E_{\mathrm{inc}}}{V_0} \tag{S12}$$

and is reported as kJ L^{-1}. The energy-normalized H_2-equivalent yield was determined from:

$$Y_E = \frac{\eta_{\mathrm{H2,eq}}}{E_{\mathrm{inc}}} \tag{S13}$$

and is reported as µmol H_2,eq kJinc^{-1}. This parameter represents an energy-normalized gas-production yield and not an apparent quantum yield, because the incident photon flux and gas composition were not determined.

4) The TiO_2 inventory was determined separately for each catalyst-deployment strategy. For suspended P25, the catalyst mass was calculated from:

$$m_{\mathrm{TiO_2,susp}} = C_{\mathrm{TiO_2}} V_0 \tag{S14}$$

where C_{TiO_2} is the initial P25 concentration. For post-print and pre-cure coatings, the retained TiO_2 mass was obtained from the dry reactor mass increase after correction for the binder contribution:

$$m_{\mathrm{TiO_2,ret}} = \Delta m_{\mathrm{coat}} w_{\mathrm{TiO_2,dry}} \tag{S15}$$

where Δm_{coat} is the dry mass increase after deposition and $w_{TiO_2,dry}$ is the TiO_2 mass fraction in the dry coating formulation. For direct printing, the nominal TiO_2 inventory was estimated as:

$$m_{\mathrm{TiO_2,inc}} = w_{\mathrm{TiO_2,form}}\, m_{\mathrm{composite}} \tag{S16}$$

where $w_{\mathrm{TiO_2,form}}$ is the P25 mass fraction in the printing formulation and $m_{composite}$ is the mass of the printed TiO_2-containing composite. The directly incorporated catalyst mass represents the total nominal TiO_2 inventory and not necessarily the fraction exposed to the liquid and irradiation.

5) The TiO_2 deposition yield was calculated as:

$$Y_{dep} = \frac{m_{\mathrm{TiO_2,ret}}}{m_{\mathrm{TiO_2,applied}}}\, x\ 100, \tag{S17}$$

where $m_{\mathrm{TiO_2,applied}}$ and $m_{\mathrm{TiO_2,ret}}$ are the initially applied and gravimetrically retained TiO_2 masses, respectively.

6) For the overall comparison of the catalyst-deployment strategies, the maximum apparent volumetric productivity *($P_{V,max}$)* was identified from the corrected time-resolved productivity profile of each configuration. The mean post-maximum apparent volumetric productivity was calculated from the cumulative H_2-equivalent production generated between the configuration-specific maximum and the selected experimental endpoint:

$$\bar{P}_{\mathrm{V,post}} = \frac{\eta_{\mathrm{H_2,eq}}(t_{\mathrm{end}}) - \eta_{\mathrm{H_2,eq}}(t_{\mathrm{max}})}{V_{\mathrm{R,eff}}\,(t_{\mathrm{end}} - t_{\mathrm{max}})} \tag{S18}$$

where t_{max} is the time corresponding to the maximum apparent volumetric productivity and t_{end} is the selected endpoint of the corresponding experiment. This definition provides the mean reactor-level productivity sustained after the maximum response and accounts for the duration of the post-maximum operating interval.

7) Productivity retention was defined as:

$$R_\mathrm{P} = \frac{\bar{P}_\mathrm{V,post}}{P_\mathrm{V,max}} \times 100 \tag{S19}$$

where $P_{V,\mathrm{max}}$ is the maximum apparent volumetric productivity of the corresponding configuration and $\bar{P}_{V,\mathrm{post}}$ is its mean apparent volumetric productivity during the subsequent post-maximum operating interval.

8) The post-maximum productivity profiles were additionally normalized according to:

$$P_\mathrm{norm}(t') = \frac{P_{V,app}(t_\mathrm{max}+t')}{P_\mathrm{V,app}(t_\mathrm{max})}, \tag{S20}$$

where $t' = t - t_\mathrm{max}$. This normalization was used exclusively to compare productivity retention after the configuration-specific maximum and does not provide information on the absolute performance of the different catalyst-deployment strategies.

The catalyst inventories, geometrical descriptors, configuration-specific effective liquid volumes and radiometric descriptors used in these calculations are summarized in Table S6.

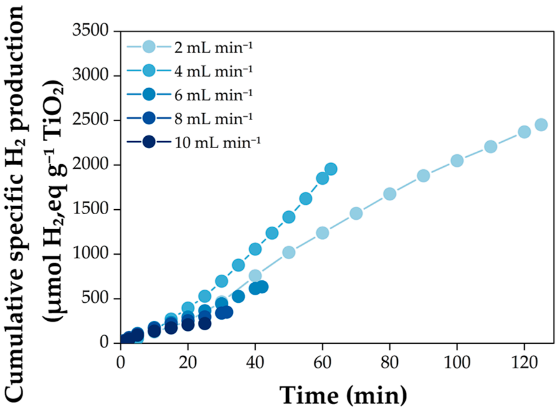

Figure S6. Time-resolved cumulative H_2-equivalent production obtained with suspended P25 TiO_2 at liquid flow rates of 2–10 mL min^{-1}. Experiments were conducted with 25 mL of 0.1 M formic acid solution, 100 mg L^{-1} P25 and 9 W UVA irradiation and were terminated after ten nominal recirculation cycles.

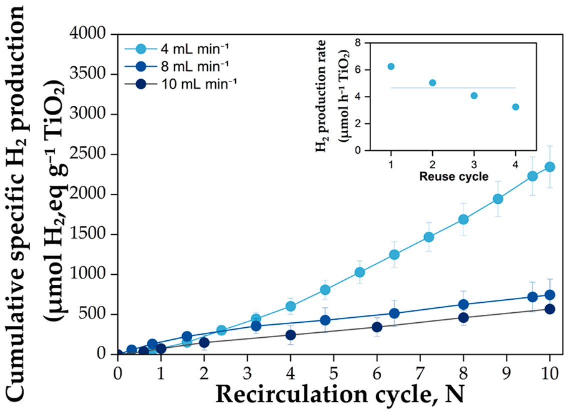

Figure S7. Short-term repeatability of the photocatalytic experiments conducted at 4, 8 and 10 mL min^{-1} using the same flat panel photoreactor. Data are presented as the means of duplicate experiments; error bars indicate the experimental range (n = 2). The inset shows the final H_2,eq production rate obtained at N = 10 for four independent runs performed at the selected flow rate of 4 mL min^{-1}; the horizontal line indicates the mean value.

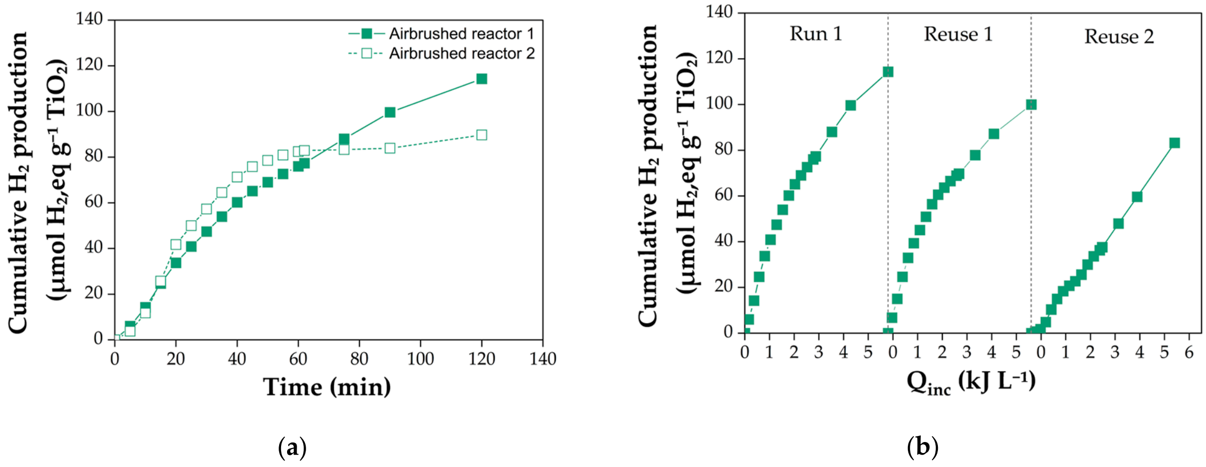

Figure S8. Assessment of the selected airbrushed P25/PARALOID™ B-72 configuration: (**a**) reactor-to-reactor reproducibility using two independently prepared reactors; and (**b**) sequential reuse of the same reactor over three runs.

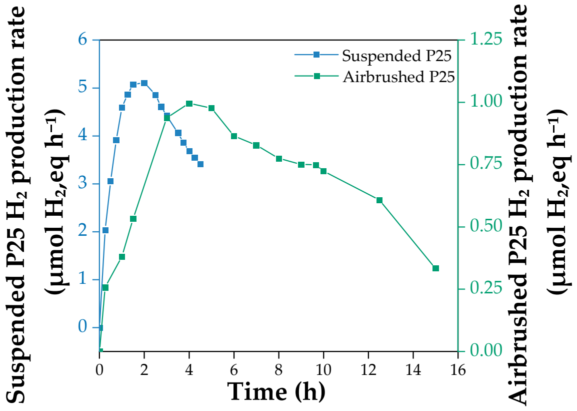

Figure S9. Experimental H_2,eq production-rate profiles of suspended P25 and the previously used airbrushed P25/PARALOID™ B-72 reactor.

*Separate ordinate axes are used because of the different absolute production-rate ranges; the corresponding post-maximum normalized profiles are compared in Figure 9C.

Table S6. Effective geometrical parameters and apparent irradiance transmission ratios of the selected reactor configurations.

**Configuration**	**δ_{eq} (mm)***	**h_{eff} (mm)**	**τ_{exp}(s)**	**$V_{R,eff}$ (mL)**	**q'_{inc} (W m^{-2})**	**q''_{out} (W m^{-2})**	**T_I (%)**
Suspended P25	—	3.00**	153.00 ± 0.28	10.23±0.02	10.3	4.9	47.6
Airbrushed P25/ PARALOID™ B-72	1.32	1.68	52.00 ± 0.57	3.44 ± 0.04	8.0	6.1	76.3
Pre-cure P25/resin	1.20	1.80	55.00 ± 0.57	3.68 ± 0.04	8.0	4.5	56.3
Directly 3D-printed P25/resin	1.77	1.23	38.00 ± 1	2.51 ± 0.07	8.9	4.1	46.1

*The equivalent layer thickness was calculated as $\delta_{eq} = h_{c,0} - h_{eff}$, using a nominal uncoated channel height of 3.00 mm. The apparent irradiance transmission ratio was calculated as $T_I = q''_{out} / q'_{inc} \times 100$. Because irradiance was measured at different positions across the reactor assembly, is used only as an operational radiometric descriptor and should not be interpreted as intrinsic material transmittance, catalyst absorptance, quantum yield or photocatalytic energy-conversion efficiency.

**For suspended P25 configuration, 3.00 mm corresponds to the nominal height of the uncoated serpentine channel; $V_{R,eff}$ was independently determined from the calibrated liquid flow rate and experimental residence time. τ_{exp} values correspond to the meaning of triplicate measurements (n = 3).

Table S7. Instrument-reported local SEM–EDS results for the post-mortem reactor surfaces.

Configuration	Elements	Ti (wt.%)*	Ti (at.%)
Suspended P25	Ti, Pd, Au	48.56	74.64
Airbrushed P25/Paraloid	Ti, Pd, Au	21.99	46.12
Pre-cure P25/resin	Ti, Pd, Au	69.57	87.25
Directly 3D-printed P25/resin	C, O, Pd, Au	n.q.	n.q.

*Values correspond to individual microscopic fields and are those reported by instrumental software. The elements included in the normalization differed among analyses: C and O were not included in the quantitative normalization of the suspended, airbrushed and pre-cure samples, whereas Ti was not detected in the selected field of the directly printed sample. Au and Pd originated from the conductive coating. Consequently, the reported Ti percentages are local and semiquantitative and must not be interpreted as total reactor composition, TiO_2 loading or directly comparable surface concentrations. *n.q.*: not quantified in the normalized EDS result.

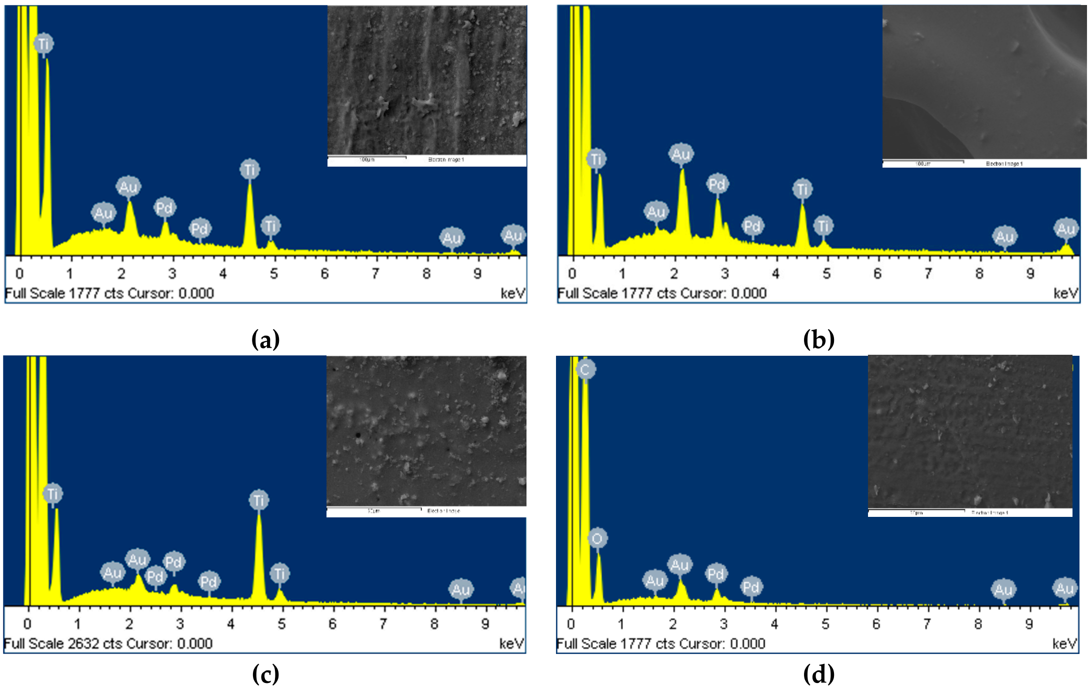

Figure S10. Representative EDS spectra of the post-mortem reactor surfaces: (**a**) uncoated reactor operated with suspended P25, (**b**) airbrushed P25/PARALOID™ B-72 coating, (**c**) pre-cure P25/resin layer and (**d**) directly 3D-printed P25/resin reactor. Au and Pd signals originate from the conductive coating applied before SEM analysis. Spectra were acquired at an accelerating voltage of 20 kV. Insets show the corresponding SEM micrographs.

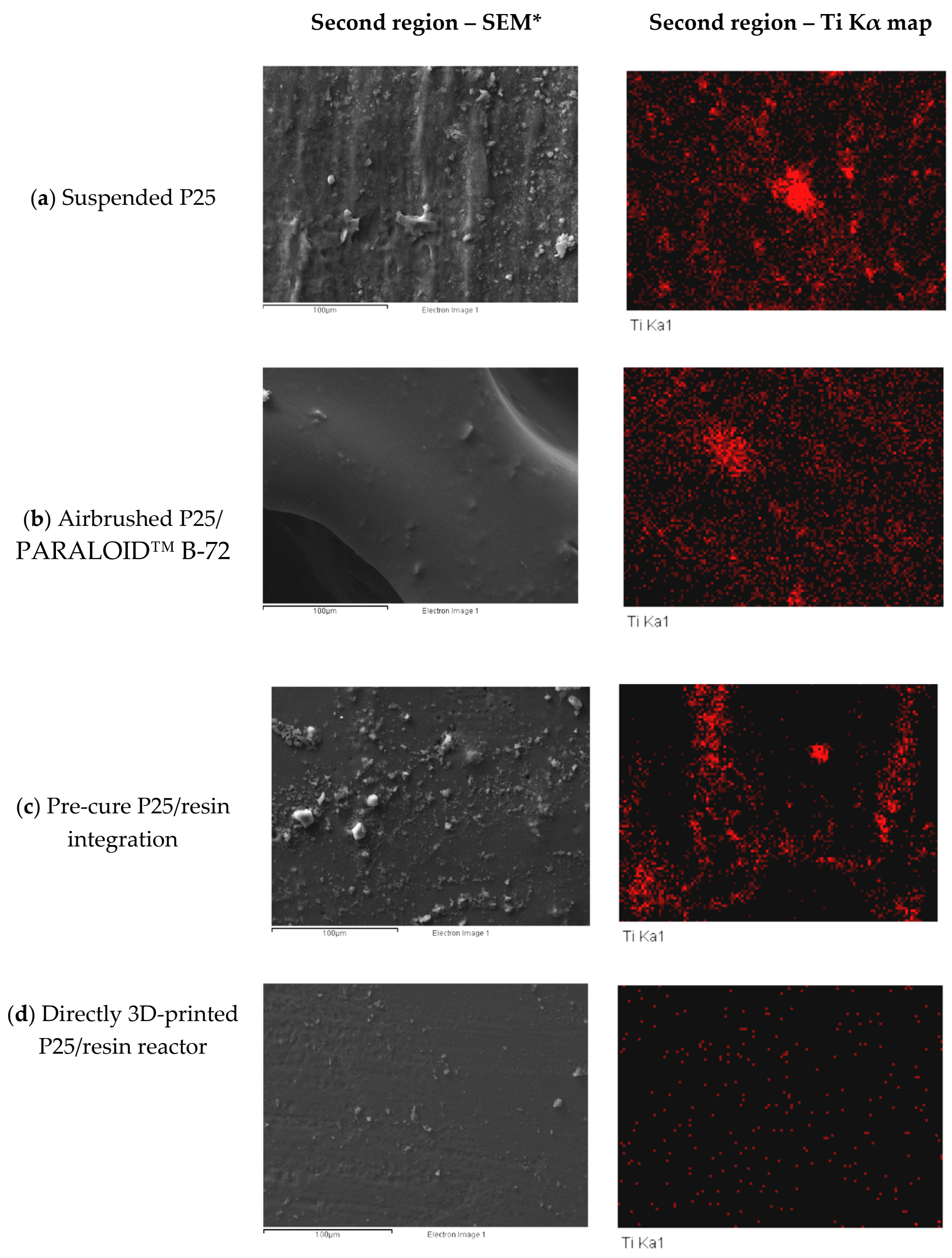

Figure S11 Additional SEM micrographs and Ti Kα elemental maps acquired from a second independent region of the post-mortem reactor surfaces: (**a**) uncoated reactor operated with suspended P25, (**b**) airbrushed P25/PARALOID™ B-72 coating, (**c**) pre-cure P25/resin integration, and (**d**) directly 3D-printed P25/resin reactor.

* These additional regions were examined to assess the spatial consistency of the local Ti distribution observed in Figure 11. SEM–EDS mapping is local and semiquantitative and should not be interpreted as a quantitative measurement of overall surface composition.

Table S8. TiO_2 inventory and mass-normalization basis of the selected catalyst-deployment configurations.

Catalyst configuration	TiO_2 content	Preparation/ formulation volume	Applied or nominal TiO_2 mass (mg)	Retained TiO_2 mass (mg)	Deposition yield, Y_{dep} (%)	Batch incorporation fraction (%)
Suspended P25	100 mg L^{-1}	25 mL	2.50	2.50	-	
Airbrushed P25/ PARALOID™ B-72	1200 mg L^{-1}	15 mL	18.00	≈ 13.05	72.50	
Pre-cure P25/resin integration	1200 mg L^{-1}	15 mL	18.00	≈ 12.08	67.11	
Directly 3D-printed TiO_2/resin reactor	1200 mg L^{-1}	≈ 300 mL	360.00	≈ 86.00[a]	-	23.9[b]

[a] Nominal TiO_2 mass estimated from the mass of the printed reactor, the TiO_2 concentration of the printing formulation and the resin density.

[b] Fraction of the TiO_2 contained in the prepared printing batch incorporated into one reactor; the remaining formulation was retained in the printer vat and therefore did not constitute a deposition loss.
